# *Trichoderma* based formulations control the wilt disease of chickpea (*Cicer arietinum* L.) caused by *Fusarium oxysporum* f. sp. *ciceris*, better when inoculated as consortia: findings from pot experiments under field conditions

**DOI:** 10.7717/peerj.17835

**Published:** 2024-08-19

**Authors:** Safeer A. Chohan, Muhammad Akbar, Umer Iqbal

**Affiliations:** 1 Department of Botany, University of Gujrat, Gujrat, Punjab, Pakistan; 2 Crop Diseases Research Institute, National Agricultural Research Centre, Islamabad, Pakistan; 3 Seed Health Lab., Plant Genetic Resources Institute, National Agricultural Research Centre, Islamabad, Pakistan

**Keywords:** *Trichoderma*, *Fusarium oxysporum*, Wilt, Disease, Chickpea, *Cicer arietinum*, Crop, Biological control

## Abstract

**Background:**

Commercial/chemical pesticides are available to control *Fusarium* wilt of chickpea, but these antifungals have numerous environmental and human health hazards. Amongst various organic alternatives, use of antagonistic fungi like *Trichoderma*, is the most promising option. Although, *Trichoderma* spp. are known to control *Fusarium* wilt in chickpea but there are no reports that indicate the biocontrol efficacy of indigenous *Trichoderma* spp. against the local pathogen, in relation to environmental conditions.

**Methods:**

In the present study, biological control activity of *Trichoderma* species formulations *viz., Trichoderma asperellum*, *Trichoderma harzianum* (strain 1), and *Trichoderma harzianum* (strain 2), either singly or in the form of consortia, was investigated against *Fusarium oxysporum* f. sp. *ciceris*, the cause of *Fusarium* wilt in chickpea, in multiyear pot trials under open field conditions. The antagonistic effect of *Trichoderma* spp. was first evaluated in *in vitro* dual culture experiments. Then the effects of *Trichoderma* as well as *F. oxysporum*, were investigated on the morphological parameters, disease incidence (DI), and disease severity (DS) of chickpea plants grown in pots.

**Results:**

In dual culture experiments, all the *Trichoderma* species effectively reduced the mycelial growth of *F. oxysporum*. *T. asperellum*, *T. harzianum* (strain 1), and *T. harzianum*(strain 2) declined the mycelial growth of *F. oxysporum*by 37.6%, 40%, and 42%. In open field pot trials, the infestation of *F. oxysporum* in chickpea plants significantly reduced the morphological growth of chickpea. However, the application of *T. asperellum*, *T. harzianum* (strain 1), and *T. harzianum* (strain 2), either singly or in the form of consortia, significantly overcome the deleterious effects of the pathogen, thereby resulted in lower DI (22.2% and 11.1%) and DS (86% and 92%), and ultimately improved the shoot length, shoot fresh weight and shoot dry weight by 69% and 72%, 67% and 73%, 68% and 75%, during the years 1 and 2, respectively, in comparison with infested control. The present study concludes the usefulness and efficacy of *Trichoderma* species in controlling wilt disease of chickpea plants under variable weather conditions.

## Introduction

Chickpea (*Cicer arietinum* L.) is an essential crop worldwide. It belongs to family fabaceae. Chickpea has annual production of over 10 million tons (Muehlbauer & Sarker, 2017). It is cultivated on over 13.5 million hectares and contributes 5.72% to total global production, annually (Jendoubi et al., 2017).

Factors responsible for less production of chickpeas include both biotic and abiotic stresses. Biotic factors include fungal, bacterial, and viral attacks (Roorkiwal et al., 2018). Numerous pathogens including fungi, nematodes, and viruses have been identified which lessen the chickpeas production. Out of these pathogens, *Fusarium* spp. significantly affect the chickpeas and cause different soil and airborne diseases (Rocha et al., 2023). *Fusarium* wilt of chickpea is the most devastating disease caused by fungal species named as *Fusarium oxysporum*, causing yield loss by 60% (Singh & Yadav, 2007). However, under favorable environmental conditions for the pathogen, yield losses have been estimated as high as 100% (Hashem, Tabassum & Abd_Allah, 2020).

Various strategies have been utilized to manage *Fusarium* wilt caused by *F. oxysporum*. Synthetic fungicides are widely used to control such devastating soil-borne pathogens. As an example, carbendazim treatment declined the *Fusarium* wilt by 24% and enhanced the yield of chickpea up to 28% (Khan et al., 2014). However, synthetic fungicides have their own limitations such as high cost, environmental pollution and adverse effects on soil microbiota, which play important roles in soil organic matter decomposition (Hoyos-Carvajal, Orduz & Bissett, 2009). Moreover, many phytopathogens have developed resistance against these fungicides as demonstrated in *Fusarium verticillioides* and *F. oxysporum* (Xu et al., 2019; Poromarto & Permatasari, 2023).

Biological control is considered an alternative method to manage the soil-borne pathogens (Biswas & Ali, 2017). Soil inhabiting filamentous fungus, *Trichoderma* species (Ascomycota) have been known for their capability as biocontrol agent (BCA) (Khan et al., 2014; Wonglom et al., 2019), against many plant pathogens (Khan et al., 2014; Hernández-Melchor, Ferrera-Cerrato & Alarcón, 2019). As an example, in a dual culture experiment, *Trichoderma* resulted in suppression of *Fusarium* colony growth by 61.1%–65.5% (Younesi et al., 2021). In another study, *Trichoderma asperellum* demonstrated the greatest degree of inhibition of mycelial growth in *Fusarium*, by 73.29% (Mishra et al., 2022). In another study, seeds treated with *T. harzianum* and *T. viride* revealed significant reduction in root rot and wilt disease caused by *Rhizoctonia solani* and *F. oxysporum,* under pot conditions (Rudresh, Shivaprakash & Prasad, 2005; Dubey et al., 2012). Nagamani, Biswas & Bhagat (2015) reported biocontrol ability of *Trichoderma* in controlling *F. oxysporum* under *in vitro* conditions and observed 81.1% efficiency of *Trichoderma*. Similarly, root rot and wilt disease caused by *R. solani* and *F. oxysporum* can be successfully controlled by the application of various strains of *Trichoderma* (Rudresh, Shivaprakash & Prasad, 2005). The introduction of *T. harzianum* propagules into the soil resulted in an increase of shoot length by 45%, under pot conditions (Yedidia et al., 2001). Similarly, *T. harzianum* enhanced the fresh shoot weight in chickpea plants by 27.3%, as compared to positive control, in pot experiment (Siddiqui & Singh, 2004).

*T. harzianum* and *Trichoderma atroviride* are some species that have multiple interactions with plants and fungal pathogens (Woo et al., 2006). Furthermore, *Trichoderma* when develop symbiotic interaction with soil microorganisms, boost the organic matter decomposition and release more nutrients that are easily available to plants to achieve sustainable agriculture by enhancing growth, productivity, and yield of crops (Ruiz-Cisneros et al., 2018; Sharma & Sharma, 2020; Turkan et al., 2023). Different species of *Trichoderma* are known for their ability to control plant diseases, enhancement of the plant growth by modifying the rhizosphere and also by activating the defense mechanisms in plants (Chaverri, Gazis & Samuels, 2011; Keswani et al., 2016). Biocontrol efficacy of *Trichoderma* depends upon several abiotic conditions, such as soil pH, water holding capacity, and soil and plant root zone temperature. During myco-parasitic mechanism, *Trichoderma* species secrete different enzymes like glucanase, chitinase, and protease that devastate the fungal cell wall and reduce the disease severity (Mukhopadhyay & Kumar, 2020).

As there are number of reports regarding bio-efficacy of *Trichoderma* in controlling plant diseases, but such reports are missing for indigenous *Trichoderma* spp. against the local pathogen. For an effective biocontrol agent (BCA), it is recommended to search the BCA from indigenous environmental niches of the fungal pathogens, as they could be more effective than exotic BCA (Yazid et al., 2023), and could reduce the risk of non target effects on the ecosystem (De Clercq, Mason & Babendreier, 2011). Although field trials are preferred methods to evaluate the crop behavior, studies suggested that outdoor pot trials are better way to evaluate the crop behavior as compared to pot trials conducted under glasshouse and are therefore, a preferred alternative if field trials are not feasible (Pastor, Palacios & Torres, 2023). Studies conducted under controlled conditions like *in vitro* or glass house conditions for improving agricultural applications is a central matter of debate, demanding a holistic approach (Nelissen, Moloney & Inzè, 2014). Therefore, the present study was designed to examine the effects of indigenous *Trichoderma* spp. as biocontrol agent against chickpea *Fusarium* wilt in multiyear outdoor pot trials, in relation to weather conditions, not reported earlier.

## Materials & Methods

### Collection of the pathogen, *Fusarium oxysporum* and *Trichoderma* spp.

Chickpea growing fields in District Chakwal, Punjab, Pakistan were surveyed to find *Fusarium* wilted chickpea plants and collection of soil samples for the isolation of indigenous *Trichoderma* spp. Three cm of the upper soil was discarded and five sub-samples were taken randomly at a depth of 20 cm and put into sterilized polyethylene bags and were brought to the laboratory for the isolation of indigenous *Trichoderma* spp. The soil samples from each site were pooled together to give one composite sample for each location (Ru & Di, 2012). *Trichoderma* spp. were isolated from sampled soil on potato dextrose agar (PDA) by serial dilution technique and the plates were kept at 26 °C for 4 days. The fungal colonies were purified and kept at 26 °C for 7–8 days. The cultures were maintained on PDA slants (Khandelwal et al., 2012). *F. oxysporum* was isolated from diseased chickpea plant and *Trichoderma asperellum* was isolated from the rhizosphere of diseased chickpea plant at location 1 (Morat), while *Trichoderma harzianum* (strain 1) and *Trichoderma harzianum* (strain 2) were isolated from the soil of location 2 (Dhok Jamal). These locations were ≈ 2.1 km apart from each other.

### Media preparation

Glass flasks were washed with tap water and rinsed thrice with distilled water. Peeled potato slices (200 g) were boiled in 500 mL dH2O for 30 min, then filtered through muslin cloth with the extract saved. A total of 20 g dextrose and 20 g agar were added in potato extract and mixed properly to avoid clot formation. Total volume of solution was made up to 1 L by adding distilled water. Media were sterilized for 20 min at 121 °C. Antibiotic streptomycin was added in the media to prevent bacterial growth and the medium was poured in sterilized glass Petri plates.

### Isolation of pathogen (*Fusarium*) from infected chickpea roots

Infected chickpea roots with symptoms of the wilt disease were sectioned (0.5–1.0 cm), washed with tap water, surface sterilized with clorox (NaOCl) for 5 min, rinsed three times with sterilized dH_2_O and dried on sterilized filter papers. These root pieces were plated at the rate of five pieces/Petri dish on the PDA medium supplemented with chloramphenicol (0.05 g/L) and incubated at 26 °C for 7 days. The isolated fungi were further sub-cultured on PDA. Pure isolates were observed for their growth patterns and pigmentation on the adverse side of the agar plates. Further microscopic examination was carried out for mycelia and conidial structures using pure culture of *F. oxysporum.* Pure cultures of the isolated fungi were transferred to PDA slants and stored in a refrigerator at 4 °C. Temporary slide mounts were prepared to confirm identity (Mohamed & Haggag, 2006).

### Isolation of *Trichoderma* spp. from soil through serial dilution method

Stock solution of sample was prepared by dissolving 1 g of soil sample into 10 mL of dH_2_O. Serial dilutions of samples were prepared at 10^−1^, 10^−2^, 10^−3^ up to 10^−7^. A total of 100 microliters of 10^−3^ of the prepared dilution was spread uniformly on the PDA in a Petri dish with the help of a glass spreader. Plates were covered with parafilm to avoid microbial contamination and placed in an incubator at 26 °C for 3 days. After 3 days, fungal growth was observed on PDA plates (Gil, Pastor & March, 2009).

### Purification of fungal spp.

Different fungal cultures were taken from actively growing ends with inoculating needle. Then these fungal cultures were inoculated on to fresh media plates. Petri plates were closed with parafilm and labeled. Plates were incubated in growth chamber and the fungal growth was checked over time.

### Identification of *Trichoderma* spp. and *F. oxysporum* on morphological basis

Pure cultures of *Trichoderma* isolates and *F. oxysporum* were observed for morphological traits *e.g.*, shape and color of colony, spore size, shape and color of conidia and conidiophores, as well as structure and branching of mycelia, based on morphological descriptions (Watanabe, 2010).

### Molecular identification of fungal species genomic DNA extraction and PCR of ITS of rDNA

DNA extraction and purification was done by following the procedure as described by (Lacap, Hyde & Liew, 2003; Ghosh & Pal, 2017). The internal transcribed spacer (ITS) regions were amplified by PCR using DNA amplification reagent kit manual (GeNei) with fungal specific forward primer ITS-1 F (Gardes & Bruns, 1993) and the reverse primer ITS-4 (White et al., 1990). PCR was done by adopting the protocol as described by Ghosh & Pal (2017), modified from (Gardes & Bruns, 1993). Amplified products were analyzed on 1% agarose gel containing 12 µL of ethidium bromide in 0.5 X Tris-borate EDTA (TBE) buffer. The purified PCR products were sequenced and obtained sequences were subjected to BLAST (https://www.ncbi.nlm.nih.gov/), and were compared with fungal sequences available at NCBI. The sequences were further manually edited and aligned using CLUSTAL W program in MEGA 11 for phylogenetic analysis and to study the diversity among tested isolates.

### Dual culture experiments

The *Trichoderma* spp. were initially assessed for their antagonistic activity against *F. oxysporum* by *in vitro* dual culture technique. A 5 mm diameter mycelial disc from the margins of the 7 days old culture of *Trichoderma* spp. and the *F. oxysporum* were placed on the opposite of the plate at equal distance. The Petri plates for each test isolate were arranged in a completely randomized design (CRD) with three replicas and incubated at 25 ± 1 °C for 7 days. Radial growth was measured after 7 days of the incubation period and % age inhibition was determined as follows;

*L* = [(*C* − *T*)/*C*] × 100

where L = inhibition of mycelial growth; C = growth measurement of the pathogen in control and T = growth of the pathogen in the presence of *Trichoderma* (Wonglom et al., 2019).

### Mass culturing of *Fusarium oxysporum* and *Trichoderma* spp.

For mass culturing of *F. oxysporum*, sorghum seeds were autoclaved in heat resistant polythene bags and were inoculated with spores of *F. oxysporum,* under aseptic conditions, in a laminar air flow cabinet. These bags were incubated at 26 ± 2 °C for ten days until all the grains were fully covered with fungal spores/mycelia. After 10 days, grains were air dried and ground to powder.

### Preparation of carrier based *Trichoderma* formulations and *Fusarium* inocula

Mass production of *Trichoderma* spp. included talc based formulation. In talc based formulation, PDA was used for initial growth of *Trichoderma* spp. in Petri plates. A total of 1,000 mL PDA was prepared in conical flask. Flask was plugged with cotton and covered with aluminum foil to avoid air-borne contamination & sterilized by autoclave at 15 lbs (121 °C temperature) for 15 min. PDA medium was poured in Petri dishes and allowed to solidify. *Trichoderma* spores were transferred in Petri plates and incubated at 25 ± 2 °C for 6 days. When *Trichoderma* covered the whole plate, then spore suspension was prepared. One mL of dH_2_O was added in *Trichoderma* culture with the help of a pipette. *Trichoderma* spores were suspended in 100 mL distilled water. After washing of chickpea seeds, these seeds were soaked in 2% sucrose solution for 6 hrs (Batta, 2004). Then extra sucrose solution was removed from chickpea seeds. These seeds were packed within polypropylene bags and sealed. PP-bags were autoclaved at 15 lbs (121 °C) for 15 min. After sterilization, one mL spore suspension was added on autoclaved chickpea seeds and incubated at 25 ± 2 °C for 15 days. After fifteen days, *Trichoderma* impregnated chickpea seeds were ground in a mixer. *Fusarium* inoculum was prepared in the same manner as described for *Trichoderma*, except that *Fusarium* mass multiplication was made on sorghum seeds (Shanmugam, Chugh & Sharma, 2015).

A total of 2 gm culture of each isolate was mixed well with 100 mL dH_2_O and filtered through muslin cloth. Few drops of Tween 20 (Alkest TW 20) were added in the spore suspension as a wetting agent (Jamil et al., 2010). Spore concentration was measured/gram of chickpeas using hemocytometer. Then the required quantities of chickpeas with *Trichoderma* inoculum were added into 1 kg sterilized talc [Mg_3_Si_4_O_10_(OH)_2_] (Batch # S.15442; Rurka Export China Food and Pharma Grade Osmanthus Brand), and 5 gm of carboxymethyl cellulose was applied to seeds and mixed thoroughly to get fine coating on seeds. The treated seeds were spread over blotter paper and air dried (Pandey, Gohel & Jaisani, 2017). The product was packed in PP-bags and sealed. The final colony forming units (CFU) in all *Trichoderma* spp. were adjusted at 1.5 × 10^7^ CFU/g of product, while, the final CFU in *F. oxysporum* was adjusted at 1.2 × 10^5^ CFU/g of the product. Fine coated seeds were sown in each sterilized pot (Bhagat & Pan, 2011).

### Pot experiments

Pot experiments were performed to check the efficacy of *Trichoderma* as biocontrol agent against *Fusarium* wilt. Chickpea susceptible variety (Pb-91) (Rashid et al., 2013) was chosen as a test crop. Certified seeds of chickpea variety were purchased from the local market.

### Preparation and sterilization of soil mixture

Silty loam soil collected from a field at National Agricultural Research Centre (NARC) Islamabad, Pakistan was passed through a sieve. The soil and river sand were mixed in a ratio of 4:1 and filled in sacks and autoclaved. Sterilized earthen pots were filled with 1.5 kg of soil (Siddiqui & Singh, 2004).

### Raising and maintenance of chickpea plants in pots

Chickpea seeds were disinfected with 0.1% mercuric chloride and rinsed thrice with autoclaved water. Five seeds were sown (during the month of November each year) into each pot at equal distance from each other. Thinning was carried out after successful emergence of seedlings to three chickpea plants per pot.

Following treatments were investigated in pot experiments;

**T1** = (Non-infested control) without *Trichoderma* and *Fusarium*

**T2** = (Infested control) *Fusarium* (1.2 × 10^5^ CFU/g of product), 10 g

**T3** = *Fusarium* (1.2 ×10^5^ CFU/g of product), 10 g + *Trichoderma asperellum* (1.5 × 10^7^ CFU/g of product) 8 g (concentration 1)

**T4** = *Fusarium* (1.2 × 10^5^ CFU/g of product), 10 g + *Trichoderma asperellum* (1.5 × 10^7^ CFU/g of product) 12 g (concentration 2)

**T5** = *Fusarium* (1.2 ×10^5^ CFU/g of product), 10 g + *Trichoderma harzianum* (strain 1) (1.5 × 10^7^ CFU/g of product) 8 g (concentration 1)

**T6** = *Fusarium* (1.2 × 10^5^ CFU/g of product), 10 g + *T. harzianum* (strain 1) (1.5 × 10^7^ CFU/g of product) 12 g (concentration 2)

**T7** = *Fusarium* (1.2 × 10^5^ CFU/g of product), 10 g + *Trichoderma harzianum* (strain 2) (1.5 × 10^7^ CFU/g of product) 8 g (concentration 1)

**T8** = *Fusarium* (1.2 × 10^5^ CFU/g of product), 10 g + *T. harzianum* (strain 2) (1.5 × 10^7^ CFU/g of product) 12 g (concentration 2)

**T9** = *Fusarium* (1.2 × 10^5^ CFU/g of product), 10 g *+ T. asperellum, T. harzianum* (strain 1), *T. harzianum* (strain 2) consortium (1.5 × 10^7^ CFU/g of product) 2.66 g of product of each *Trichoderma* (concentration 1)

**T10** = *Fusarium* (1.2 × 10^5^ CFU/g of product), 10 g + *T. asperellum, T. harzianum* (strain 1), *T. harzianum* (strain 2) consortium (1.5 × 10^7^ CFU/g of product) 4 g of product of each *Trichoderma* (concentration 2)

Experimental design was CRD. Pot experiments were executed in two consecutive seasons (2020–2021 & 2021–2022). Each treatment was replicated three times. Field related experiments were approved by Crop Diseases Research Institute (CDRI), National Agricultural Research Centre, Islamabad, vide Dy No. 10712.

### Disease measurements, disease incidence and severity

In case of disease measurements in pot experiments, disease incidence (DI) and disease severity (DS) were determined (Khan, Khan & Mohiddin, 2004; Nandeesha & Huilgol, 2021).

Following formula was used for determination of DI and DS. For DS, 0–5 scale was adopted; 0 = no wilt, 1 = 1–20%, 2 = 21–40%, 3 = 41–60%, 4 = 61–80%, & 5 = 81–100% (Khan, Khan & Mohiddin, 2004; Jamil & Ashraf, 2020). These data in pots were recorded 68 days after sowing (DAS), in the years 1 & 2.

Wilt incidence (%) = $ \frac{\text{Total}~\text{number}~\text{of}~\text{wilted}~\text{plants}}{\text{Total}~\text{number}~\text{of}~\text{plants}~\text{observed}} \times 100$

Disease severity = Number of branches, twigs, or leaves showing wilt symptoms/total number of branches, twigs, or leaves.

### Harvesting and data collection in pot experiments

Pot data were taken after 101 DAS, during the month of February each year. Following morphological parameters were recorded in pot experiments; shoot length, shoot fresh and dry weights. In case of disease measurements, DI and DS were determined (Dubey, Suresh & Singh, 2007).

### Soil analyses

Soil analyses of chickpea growing fields used to isolate *Fusarium* and indigenous *Trichoderma* spp. and of pot soils were carried out by following the procedures as described by Estefan, Sommer & Ryan (2013) (Table 1).

**Table 1:** Soil composition of chickpea growing fields used to isolate *Fusarium* and indigenous *Trichoderma* spp. and of pot soils.

**Treatments**	**Soil texture**	**pH**	**SOM**	**NO_3_-N**	**Available P**	**Available K**	**Zn**	**Fe**	**Cu**
**Location 1**	Sandy loam	7.50 ± 0.14b	0.48 ± 0.04c	3.5 ± 0.1a	5.2 ± 0.35b	92 ± 3.6b	0.91 ± 0.03a	6.03 ± 0.2c	1.21 ± 0.04a
**Location 2**	Sandy loam	7.9 ± 0.4a	0.64 ± 0.03a	2.1 ± 0.1d	6.5 ± 0.37a	101 ± 2.8a	0.94 ± 0.07a	5 ± 0.1d	1.03 ± 0.06b
**Pot year 1**	Sandy loam	7.4 ± 0.11b	0.55 ± 0.02b	2.65 ± 0.05b	6.14 ± 0.2a	79.3 ± 1.5c	0.66 ± 0.03b	9.7 ± 0.7a	0.37 ± 0.01d
**Pot year 2**	Sandy loam	7.51 ± 0.09b	0.6 ± 0.03ab	2.33 ± 0.06c	5.6 ± 0.1b	80 ± 1.3c	0.62 ± 0.02b	8.8 ± 0.3b	0.44 ± 0.02c

**Notes.**

Values indicate averages of three repetitions ± standard deviation. In each column, averages with common alphabets do not differ at *P* = 5%, as computed by Fisher’s LSD test using Minitab 20.2. Significance was determined within each column.

SOM = soil organic matter%; Nitrate-nitrogen (NO_3_-N) mg/kg; Available phosphorus (P) mg/kg; Available potassium (K) mg/kg; Zinc (Zn) mg/kg; Iron (Fe) mg/kg; Copper (Cu) mg/kg

### Environmental data

Environmental data were collected from the Pakistan Meteorological Department, Islamabad, Pakistan (Table 2).

**Table 2:** Average rainfall (mm) and temperature (degree celsius) data.

**Rainfall**				
**Years**	**Nov**	**Dec**	**Jan**	**Feb**
**20–21**	92.83	21.21	11.73	8.02
**21–22**	0.01	9.82	174.52	19.22
**Max-Temperature**				
**Years**	**Nov**	**Dec**	**Jan**	**Feb**
**20–21**	23.1	18.9	19.2	24
**21–22**	25.2	19.9	16.3	20
**Min-Temperature**				
**Years**	**Nov**	**Dec**	**Jan**	**Feb**
**20–21**	7.1	4.2	2.6	6.8
**21–22**	6.9	2.9	4.7	6.6

### Confirmation of Koch’s postulates

To fulfill Koch’s postulates, healthy chickpea plants were inoculated with *F. oxysporum* grown on a culture that had been isolated from *Fusarium* wilt affected chickpea root. Then the plant was studied for disease symptoms and its comparison to the infected plants that had been used for the isolation of *F. oxysporum.*

### Statistical analyses

All the data were analyzed by ANOVA, and after performing ANOVA, Fisher’s LSD test was performed at *P* = 5%, using Minitab 20.2.

## Results

### Morphological identification of isolated *Fusarium* and *Trichoderma* strains

*F. oxysporum* showed two types of conidia; macroconidia were boat shaped and four celled, while microconidia were ellipsoidal shaped and one celled. Chlamydospores were brown in color and globose shaped. Colony was white in color. Microconidia were with size of 6.1 µm × 3.2 µm. While, macroconidia were sickle shaped with an average size of 30.7 µm × 3.4 µm.

*T. harzianum* showed conidiophores and hyaline phialides were short and thick. Conidia were ovate shaped and one-celled. Conidiophore size was 78 µm, while conidia were 2.4 µm in diameter. Colony was dark green in color on growth medium. On the other hand, *T. asperellum* had slightly ovoidal shaped conidia, having size of 2.91 µm × 2.37 µm. Conidia were one-celled.

### Phylogenetic analysis of *Trichoderma* spp.

**Figure 1:**
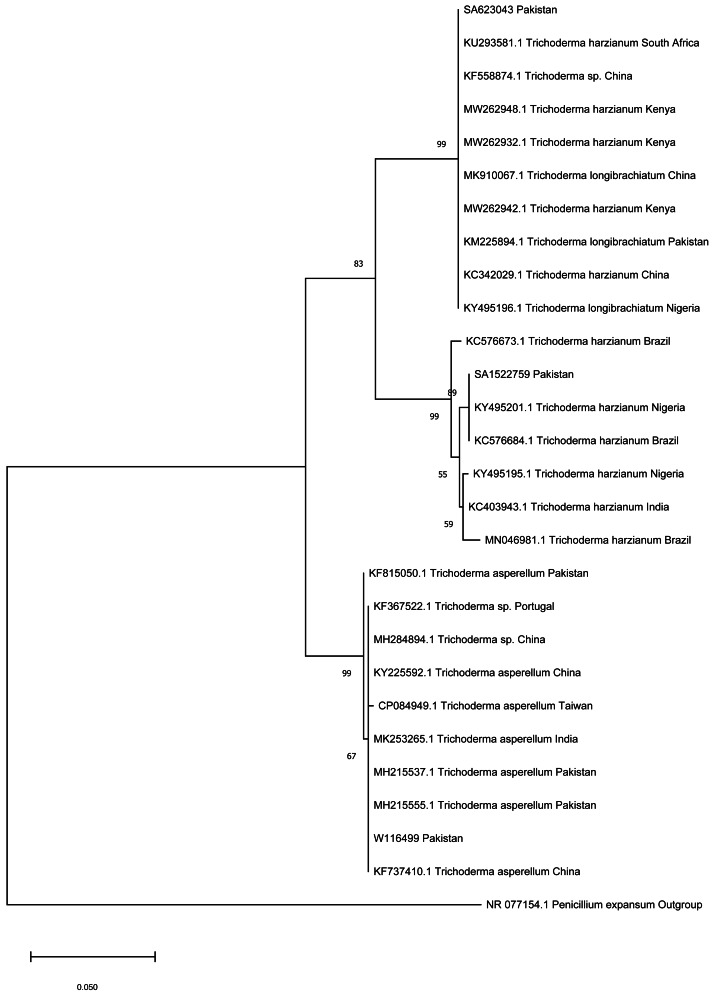
Phylogenetic tree of *Trichoderma* isolates.

Phylogenetic analysis had shown that the three isolates of the Pakistani *Trichoderma* collected for this study clustered in three clades with reference sequences from different regions of the world. The *Trichoderma* sp. (SA623043) reported in this study clustered with a sequence of *Trichoderma* from Pakistan, China, and Africa with 99% bootstrap value. The *Trichoderma* sp. (SA1522759) reported in this study clustered with two sequences of *Trichoderma* sp. from Nigeria and Brazil that was part of another clade with sequences from Brazil and Nigeria. The third isolate of *Trichoderma* sp. (W116499) reported in this study clustered with number of sequences of *Trichoderma* from Pakistan, China, Portugal, and Taiwan with 99% bootstrap value. The phylogenetic framework based on ITS sequences clarified the *T. harzianum,* although belonging to the same species, yet they phylogenetically belong to different clades (Fig. 1).

### Phylogenetic analysis of *F. oxysporum* f. sp. *ciceris*

Phylogenetic analysis had shown that the isolate of the Pakistani *F. oxysporum* f. sp. *ciceris* analysed in the present study grouped in the major clade with reference sequences from Pakistan, India, Egypt, Turkey, and China with 48% bootstrap value. The *F. oxysporum* f. sp. *ciceris* (OR808009) reported in this study clustered in a sub-clade with a sequence of *Fusarium* from India and Egypt.

**Figure 2:**
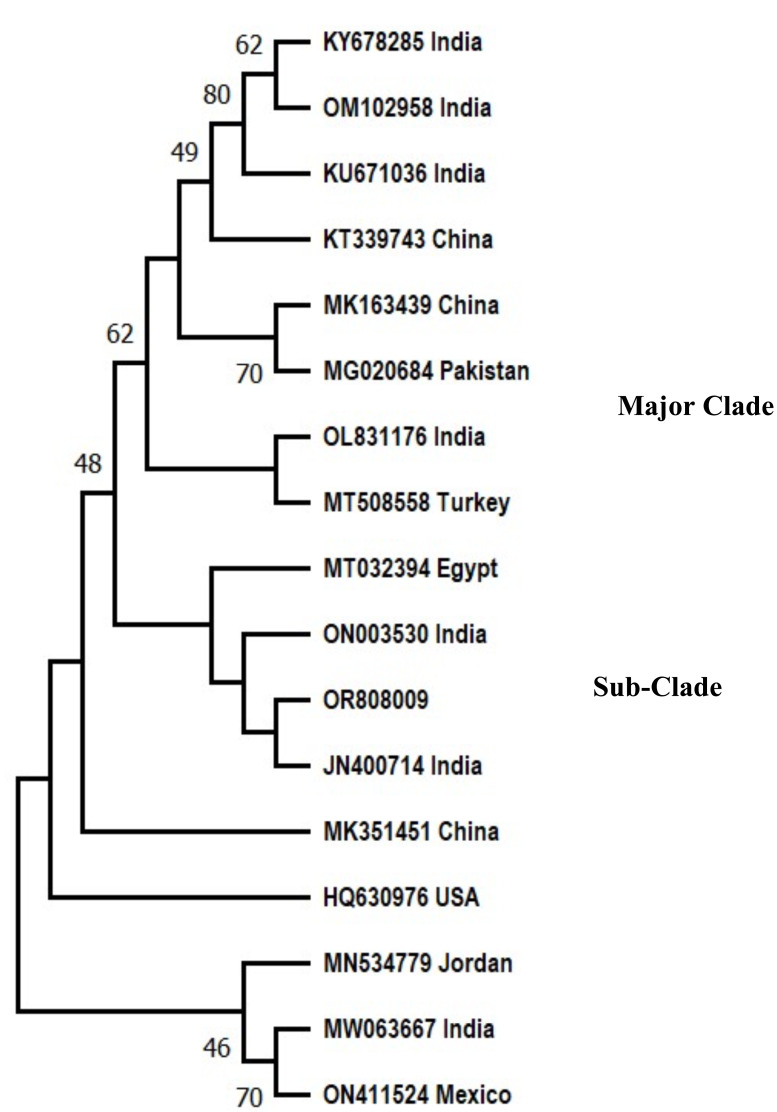
Phylogenetic tree of *Fusarium oxysporum* f. sp. *ciceris*.

The evolutionary history was deduced with Neighbor-Joining technique (Saitou & Nei, 1987). The % age of replica trees in which the associated taxa clustered together in the bootstrap test (500 replicas) are shown next to the branches (Felsenstein, 1985). The evolutionary distances were computed by the Maximum Composite Likelihood technique (Tamura, Nei & Kumar, 2004) and are in the units of number of base substitutions per site. This analysis involved 17 nucleotide sequences including nucleotide sequence (OR808009) reported in present study. All obscure positions were removed for each sequence pair (pairwise deletion option). There were a total of 1,244 positions in the final dataset. Evolutionary analyses were performed on MEGA 11 (Tamura, Stecher & Kumar, 2021) (Fig. 2).

### Effect of various *Trichoderma* spp. on *Fusarium* in dual culture experiments

In dual culture experiments, *T. asperellum* inhibited the *F. oxysporum* f. sp. *ciceris* by 37.6%, while, *T. harzianum* strains 1 and 2 significantly inhibited the radial/mycelial growth of *F. oxysporum* by 40% and 42%, respectively (Table 3).

**Table 3:** Percentage inhibition of *F. oxysporum* f. sp. *ciceris* in dual culture experiments.

**Serial no.**	**Treatments**	**Measurements of colonies in centimetres**			**% age inhibition of** ***Fusarium***
		Length	Width	Average	
**1-**	*Fusarium* in control	4.4 ± 0.79a	6.5 ± 0.76a	5.45 ± 0.62a	–
**2-**	*Fusarium* with *Trichoderma asperellum*	2.3 ± 0.50b	4.5 ± 0.53b	3.4 ± 0.33b	37.6
**3-**	*Fusarium* with *Trichoderma harzianum* I	2.4 ± 0.40b	4.1 ± 0.40b	3.25 ± 0.20b	40
**4-**	*Fusarium* with *Trichoderma harzianum* II	2 ± 0.46b	4.3 ± 0.46b	3.15 ± 0.46b	42

**Notes.**

Values are means of 3 replicates ± standard deviation. Standard deviation values were rounded off to 2^nd^ decimal. Values sharing same letter do not differ at *P* = 5%, as computed by Fisher’s LSD test using Minitab 20.2. *Fusarium* stands for *F. oxysporum* f. sp. *ciceris*. Significance was determined within each column

### Effects on shoot length, shoot fresh weight, and shoot dry weight of chickpea plant in pot trials (year 1)

**Figure 3:**
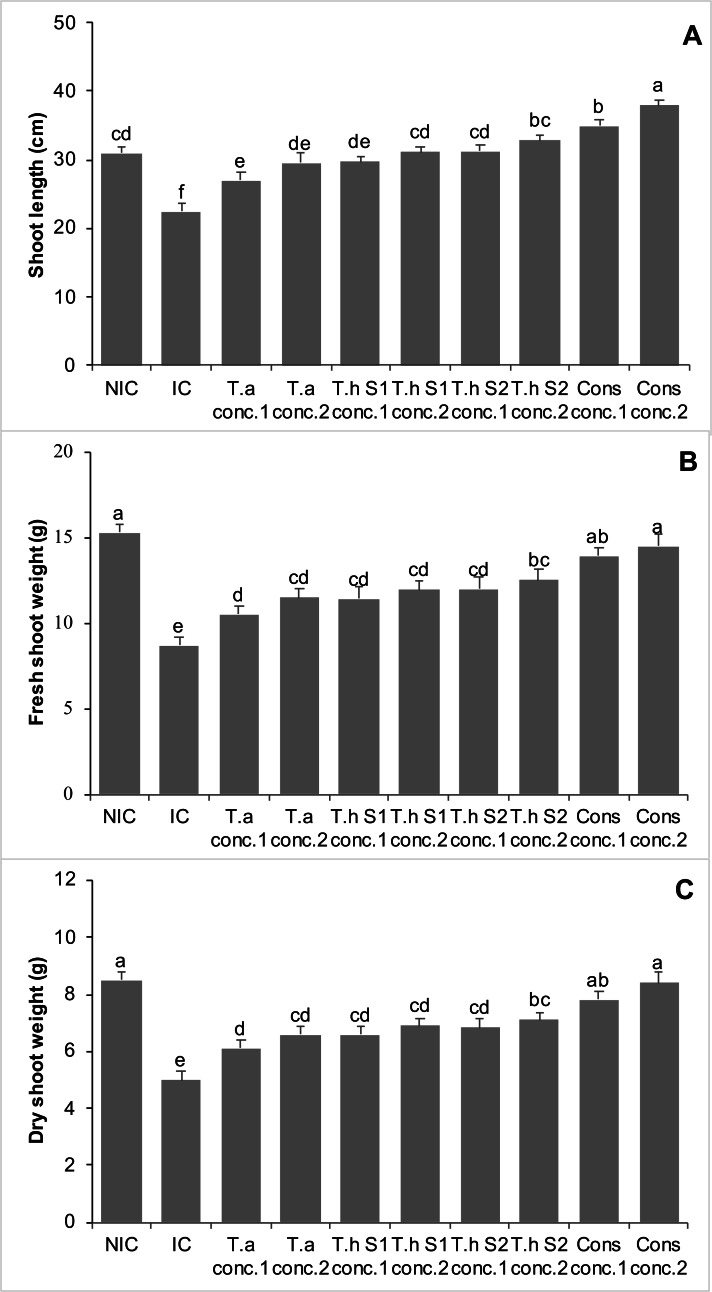
Effect of treatments on shoot length (A), shoot fresh weight (B), shoot dry weight (C) of chickpea in pot trials (Year 1). Bars with common alphabets do not differ at *P* = 5%, as computed by Fisher’s LSD test using Minitab 20.2. Y-error bars depict the standard error of three repetitions. NIC = Non infested control; IC = Infested control; T.a = *Trichoderma asperellum,* T.h S1 = *Trichoderma harzianum* strain 1; T.h S2 =* Trichoderma harzianum* strain 2; Cons = consortium of T.a, T.h S1 & T.h S2; conc. = concentration.

The *Fusarium* infested chickpea plants (infested control, IC) showed a 27% decrease in shoot length as compared to non-infested control (NIC). Treatments with *T. asperellum* (T.a) concentration 1 and concentration 2, *T. harzianum* strain 1 (Th S1), concentration 1 and concentration 2, *T. harzianum* strain 2 (Th S2) concentration 1 and concentration 2, and consortium of all these *Trichoderma* species (concentration 1&2), significantly increased the shoot length of chickpea plants by 20% and 31%, 32% and 39%, 39% and 46%, 56% and 69%, respectively, over IC. Moreover, consortium of all these *Trichoderma* species at concentrations 1 and 2, significantly enhanced the shoot length of chickpea plants by 13% and 23%, respectively, in comparison with NIC (Fig. 3A).

The chickpea plants that were infested with *Fusarium* experienced a 43% decrease in the shoot fresh weight as compared to NIC. However, all treatments involving *Trichoderma* showed a significant increase in shoot fresh weight, in comparison with IC, effectively counteracting the negative effects of the *F. oxysporum* pathogen. The effectiveness of *Trichoderma* in controlling *F. oxysporum* was found to depend on the dosage. Treatments with T.a, Th S1, Th S2, and a combination of these *Trichoderma* species, all significantly increased the shoot fresh weight of chickpea plants. These increases were 21% and 32%, 31% and 37%, 38% and 44%, and 60% and 67% at concentrations 1 and 2, respectively, compared to the IC (Fig. 3B).

IC of chickpea plants, affected by *Fusarium*, exhibited a 41% decrease in shoot dry weight, when compared to the NIC. However, all treatments involving *Trichoderma* showed a significant increase in shoot dry weight, effectively mitigating the negative effects of the pathogen, *F. oxysporum*. The efficacy of *Trichoderma* in controlling *F. oxysporum* was found to be dependent on the dosage. Treatments with T.a, Th S1, Th S2, and a consortium of these *Trichoderma* species significantly increased the shoot dry weight of chickpea plants. The increases were 22% and 32%, 32% and 38%, 37% and 42%, and 56% and 68% at concentrations 1 and 2, respectively, compared to the IC. Furthermore, the consortium of all these *Trichoderma* species at concentrations 1 and 2 showed non-significant differences when compared to the NIC (Fig. 3C).

### Effect on disease incidence and disease severity of chickpea plants in pot trials (year 1)

There was 88.9% disease incidence (DI) in *Fusarium* infested chickpea plants, as compared to NIC. All the treatments with *Trichoderma* demonstrated a significant decrease in DI of chickpea plants. Treatments with T.a, Th S1, Th S2, and consortium of all these *Trichoderma* spp. significantly decreased the DI of chickpea plants by 77.8% and 66.7%, 66.7%, 55.6% and 44.4%, 33.3%, 33.3% and 22.2%, at concentrations 1 and 2, respectively, over IC. Moreover, consortium of all these *Trichoderma* spp. at concentrations 1 and 2, significantly reduced the DI on chickpea plants by 33.3% and 22.2%, respectively, in comparison with infested control (IC).

In year 1, IC had a disease severity (DS) of 4.7, whereas, there were no disease symptoms in NIC. Moreover, all the treatments using *Trichoderma* showed significant reduction on DS in the chickpea plants and this reduction in DS was dose dependent. Treatments using T.a, Th S1, Th S2, and a combination of all these *Trichoderma*, all significantly decreased the DS in the chickpea plants. The reductions in DS were 21% and 23%, 29%, 31%, and 40%, and 47%, 71%, and 86% at concentration 1 and concentration 2, respectively, compared to the IC (Table 4).

**Table 4:** Disease incidence (DI) and disease severity (DS) on chickpea plants in pots (year 1 and year 2).

**Treatments**	**Year 1** (2020–2021)				**Year 2** (2021–2022)			
	**DI**		**DS**		**DI**		**DS**	
		Reduction over IC (%)		Reduction over IC (%)		Reduction over IC (%)		Reduction over IC (%)
**NIC**	0 ± 0e	–	0 ± 0e	–	0 ± 0f	–	0 ± 0f	–
**IC**	88.89 ± 19.2a	0	4.67 ± 0.33a	0	77.78 ± 19.3a	0	4.11 ± 0.84a	0
**T.a conc.1**	77.78 ± 19.2ab	12.5	3.68 ± 0.86ab	21.2	66.67 ± 0ab	14.3	3.55 ± 0.38ab	13.6
**T.a conc.2**	66.67 ± 33.3a-c	25	3.58 ± 0.82ab	23.3	55.56 ± 19.2a-c	28.6	2.89 ± 0.19b	29.7
**T.h S1 conc.1**	66.67 ± 33.3a-c	25	3.33 ± 0.85b	28.7	55.56 ± 19.2a-c	28.6	2.89 ± 0.19b	29.7
**T.h S1 conc.2**	55.56 ± 19.2a-d	37.5	3.23 ± 0.68b	30.8	44.44 ± 19.2b-d	42.9	2.67 ± 0.57bc	35
**T.h S2 conc.1**	44.44 ± 19.2b-d	50	2.8 ± 0.17b	40	44.44 ± 19.2b-d	42.9	2.67 ± 0.57bc	35
**T.h S2 conc.2**	33.33 ± 0c-e	62.5	2.47 ± 0.4bc	47.1	33.33 ± 0c-e	57.1	1.78 ± 0.38cd	56.7
**Cons conc.1**	33.33 ± 33.3c-e	62.5	1.34 ± 1.35cd	71.3	22.22 ± 19.2d-f	71.4	1.33 ± 1.15d	67.6
**Cons conc.2**	22.22 ± 19.2de	75	0.67 ± 0.57de	85.7	11.11 ± 19.2ef	85.7	0.33 ± 0.57e	92

**Notes.**

NIC Non infested control IC Infested control T.a *Trichoderma asperellum* T.h S1 *Trichoderma harzianum* strain 1 T.h S2 *Trichoderma harzianum* strain 2 Cons consortium of T.a, T.h S1 & T.h S2 conc. concentration

Values indicate averages of three repetitions ± standard deviation. In each column, averages with common alphabets do not differ at *P* = 5%, as computed by Fisher’s LSD test using Minitab 20.2. Significance was determined within each column.

### Effect on disease incidence and disease severity of chickpea plants in pot trials (year 2)

There was 77.8% DI in *Fusarium* infested chickpea plants (IC), as compared to NIC. All the treatments with *Trichoderma* demonstrated a significant decrease in DI of chickpea plants. The increase in the concentration of *Trichoderma* resulted in a decrease of DI. Treatments with T.a, Th S1, Th S2, and consortium of all these *Trichoderma* species significantly decreased the DI of chickpea plants by 66.7% and 55.6%, 55.6%, 44.4% and 44.4%, 33.3%, 22.2% and 11.1%, at concentrations 1 and 2, respectively, over IC. Moreover, consortium of all these *Trichoderma* spp. at concentrations 1 and 2, significantly reduced the DI of chickpea plants by 22.2% and 11.1%, respectively, in comparison with infested control (IC).

In year 2, IC revealed DS score of 4.1. *Trichoderma* showed a significant decrease in DS caused by the pathogen in the chickpea plants. Treatments using T.a, Th S1, Th S2, and a combination of all these *Trichoderma* species significantly reduced the DS in the chickpea plants. The reductions in DS were 14% and 30%, 30%, 35%, and 35% at concentration 1, and 57%, 68%, and 92%, at concentrations 1 and 2, respectively, in comparison with IC (Table 4).

### Effects on shoot length, shoot fresh weight, and shoot dry weight of chickpea plants in pot trials (year 2)

**Figure 4:**
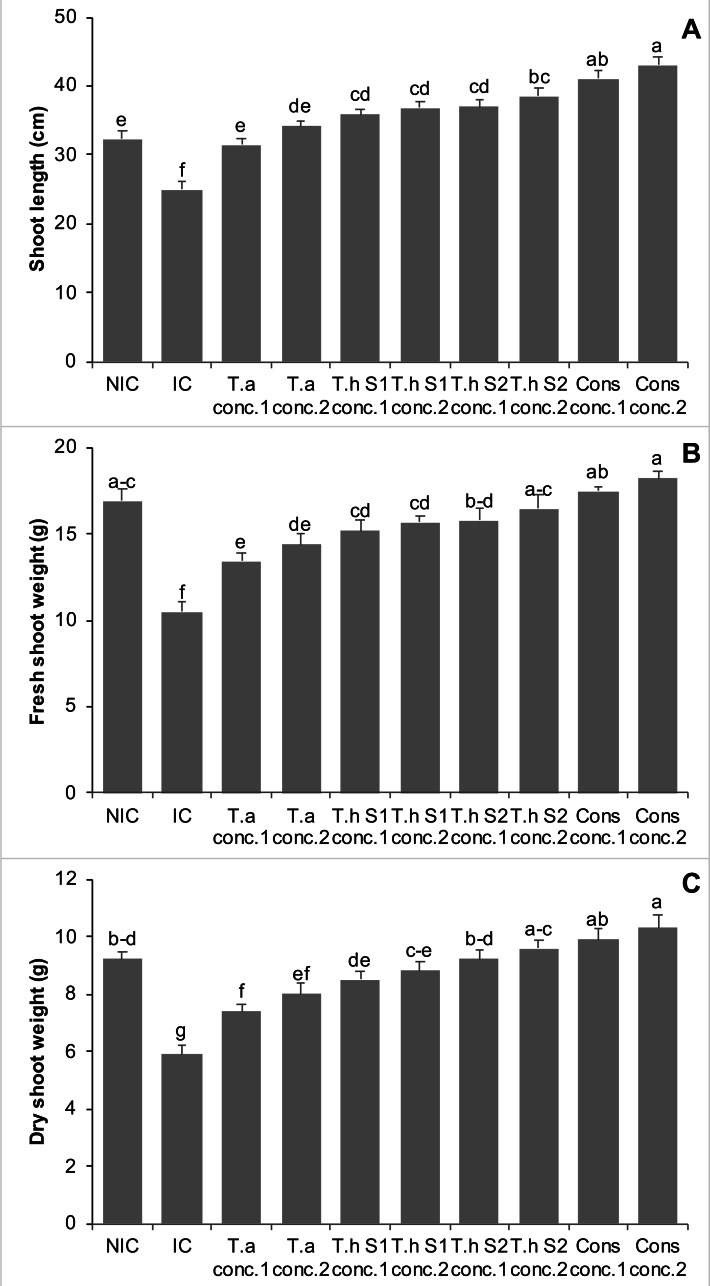
Effect of treatments on shoot length (A), shoot fresh weight (B), & shoot dry weight (C) of chickpea in pot trials (Year 2). Bars with common alphabets do not differ at *P* = 5%, as computed by Fisher’s LSD test using Minitab 20.2. Y-error bars depict the standard error of three repetitions. NIC = Non infested control; IC = Infested control; T.a = *Trichoderma asperellum,* T.h S1 = *Trichoderma harzianum* strain 1; T.h S2 =* Trichoderma harzianum* strain 2; Cons = consortium of T.a, T.h S1 & T.h S2; conc. = concentration.

In year 2 pot experiments, infested control showed a 23% decrease in shoot length as compared to NIC. All the treatments with *Trichoderma* demonstrated a significant increase in shoot length of chickpea plants, minimizing the negative effects of the pathogen, *F. oxysporum*. The effect of *Trichoderma* in controlling the *F. oxysporum* was found dose dependent. Treatments with T.a, Th S1, Th S2, and consortium of all these *Trichoderma* species significantly increased the shoot length of chickpea plants by 26% and 36%, 44% and 47%, 48% and 54%, 64% and 72%, at concentrations 1 and 2, respectively, over IC. Moreover, consortium of all these *Trichoderma* species at concentrations 1 and 2, significantly enhanced the shoot length of chickpea plants by 27% and 33%, respectively, in comparison with NIC (Fig. 4A).

The *Fusarium* infested chickpea plants revealed 38% decline in the shoot fresh weight compared to NIC. All treatments involving *Trichoderma* either alone or in the form of consortia, showed significant enhancement in shoot fresh weight of chickpea plants, indicting the beneficial effect of *Trichoderma* against *F. oxysporum*. Treatments with T.a, Th S1, Th S2, and a combination of these *Trichoderma* species, all significantly increased the shoot fresh weight of chickpea plants. The increases were 28% and 37%, 45% and 50%, 50% and 57%, and 67% and 73% at concentrations 1 and 2, respectively, compared to the IC. Furthermore, the combination of all these *Trichoderma* species at concentrations 1, 2 and Th S2 showed non-significant differences, in comparison with NIC (Fig. 4B).

*Fusarium* infection exhibited a 36% decrease in shoot dry weight of chickpea plants as compared to NIC. Treatments with T.a, Th S1, Th S2, and a consortium of these *Trichoderma* species significantly increased the shoot dry weight of chickpea plants by 25% and 36%, 44% and 49%, 56% and 63%, and 68% and 75% at concentrations 1 and 2, respectively, compared to the IC (Fig. 4C).

### Soil analyses

Soil texture was sandy loam at chickpea growing locations as well as the soil used in pot experiments. There was significant difference in the soil pH and soil organic matter (SOM) of both locations, while the pH and SOM of soil used in pot experiments in both years were non-significant, when compared with each other. NO_3_-nitrogen, available phosphorus, available potassium, iron, and copper were significantly different at both locations, except zinc. On the other hand, NO_3_-nitrogen, available phosphorus, iron, and copper were significantly different in the soils used in pots of year 1 and year 2, except available potassium and zinc. Moreover, variable results were recorded when we compare the soil properties used in pot experiments as compared with soil properties of location 1 and 2 (Table 1).

### Environmental data

Environmental data for the years 2020–2021 and 2021–2022 showed that during the critical stage (flowering stage susceptible to *Fusarium* wilt = January), there was high rainfall (Table 2).

### Confirmation of Koch’s postulates

The healthy chickpea plants inoculated with *F. oxysporum* grown on a culture that had been isolated from *Fusarium* wilt affected chickpea root in the pot trials, developed the same symptoms as inoculated initially.

## Discussion

In the present study, *Trichoderma* species significantly inhibited the mycelial growth of *F. oxysporum* f. sp. *ciceris* (FOC) in dual culture experiments. These results are consistent with the findings of Al-Surhanee (2022) who reported the 25% reduction in *F. oxysporum* in dual culture experiments. Similarly, *Trichoderma* strain ThrAN-5 showed 69% inhibition in mycelial growth of *F. oxysporum* (Bhagat & Pan, 2011). Moreover, *T. harzianum* revealed the *in vitro* bio-efficacy against different pathogens and exhibited 81% inhibition (Kumari et al., 2020). In another investigation, the *Trichoderma* isolates exhibited high competitive ability and the synthesized metabolites demonstrated inhibitory effects on the mycelial growth of *Fusarium*. *Trichoderma* isolates’ mechanisms of action is primarily linked to the generation of volatile organic compounds. Although *Trichoderma* exhibited an antagonistic effect *in vitro*, they did not demonstrate efficacy in controlling *Fusarium* or promoting chickpea growth under *in vivo* conditions (Queirozazevedo et al., 2020). But there are numerous studies that reveal the effectiveness of *Trichoderma* in controlling the pathogens and boosting the plant growth, under pot conditions.

In the present investigation, *Trichoderma* either alone or in consortia significantly declined the DI and DS in chickpea plants under multiyear pot trials. Our findings are in accordance with the results of Ramanagouda, Naik & Sharma (2022) who reported the effectiveness of *T. harzianum* against *Fusarium* wilt of chickpeas caused by FOC. In another investigation, the application of *T. harzianum* significantly reduced the DI in chickpea plants by 44%, as compared to untreated control (Nandeesha & Huilgol, 2021). The implementation of *T. harzianum* treatment resulted in significant enhancements in various plant growth parameters of chickpea plants as there was 3% increase in the plant height (Martínez-Martínez et al., 2020). In another pot experiment, *T. harzianum* increased the shoot length, shoot fresh and dry weight of chickpea plants by 46.9%, 27.3%, and 79.4%, respectively, as compared to *Fusarium* infested control (Siddiqui & Singh, 2004). In another investigation, *T. harzianum* increased the shoot height, fresh and dry weight of chickpea plants by 36.5%, 25%, and 33%, respectively, as compared to infested control pots (Meher, Singh & Sonkar, 2018). In another study, there was a significant rise in the dry weight of chickpea by 80% by inoculation of *T. harzianum* (Yedidia et al., 2001).

*Trichoderma* species are fast-growing fungi (Dutta et al., 2023), and can colonize the plant root system (Tyśkiewicz et al., 2022) and its surrounding soil (Brotman et al., 2013), competing with *Fusarium* spp. for space and nutrients (Oszust, Cybulska & Frąc, 2020). These are mycoparasites, attacking and parasitizing other fungi, including *Fusarium*, and produce the enzymes and secondary metabolites that break down the *Fusarium* cell walls and cause leakage of cellular contents (Segaran, Shankar & Sathiavelu, 2022). *Trichoderma* can induce resistance against *Fusarium* wilt (Ponsankar et al., 2023), by inhibiting the mycelial growth of fungi and by reducing the disease intensity (Sánchez-Montesinos et al., 2021). Some *Trichoderma* strains produce volatile compounds that inhibit *Fusarium* growth and directly suppress the *Fusarium* populations in the soil (Zhang et al., 2021). *Trichoderma* can enhance nutrient uptake, stress tolerance, and help the chickpea plants to withstand under *Fusarium* wilt stress and recover more effectively from infection (Singh & Vyas, 2023). In an investigation, *T. harzianum* significantly reduced the DI by 62%, as compared to positive (pathogen infested) control (Nandeesha & Huilgol, 2021). Similarly, *T. harzianum* reduced the DI by 75%, as compared to positive control (Ghosh, Banerjee & Sengupta, 2017). Other studies also reported decline in the DI by 65% and 80%, as compared to positive control, by the inoculation of *T. harzianum* (Ali & Terefe, 2021; Anwar et al., 2022). The findings of another study indicated that the application of *Trichoderma viride* to chickpea seeds *via* soil treatment resulted in the lowest incidence of wilt at 21.50%. In an investigation, variations in the effectiveness of *Trichoderma* species were recorded as the consortium of *Trichoderma virens* and *T. harzianum* showed DI by 29.4%. The consortium of *T. virens* and *T. viride* showed DI by 31.3%. While, the consortium of *T. viride* and *T. harzianum* reduced the DI by 54.9% (Tomar, Thakur & Yadav, 2022). On the other hand, the consortium of two strains of *T. harzianum* decreased the DI by 128% in tomato plants, in comparison to control (Singh et al., 2014).

In the present study, *Trichoderma* consortia significantly improved the growth of chickpea plants in pot trials. Similar findings were previously documented by Trivedi et al. (2020) when plants were treated with *T. harzianum* and reported 29% increase in shoot length, as compared to positive control. Moreover, a consortium of two *T. harzianum* significantly enhanced the tomato shoot length by 35%, as compared to negative control (Singh et al., 2014). Similarly, *T. harzianum* increased dry shoot weight by 29.7%, as compared to positive control (Khan et al., 2014). Likewise, *T. harzianum* increased dry shoot weight by 32.5%, as compared to positive control (Khan, Khan & Mohiddin, 2004). In another *in vivo* investigation, *Trichoderma cerinum* caused 13.3% and 58.3% increase in dry weight of chickpea with or without *F. oxysporum*, respectively (Khare, Kumar & Kim, 2018).

*Trichoderma* species produce different kinds of compounds and siderophores that boost the organic matter decomposition in the soil (Kubheka & Ziena, 2022). These compounds improve the solubilization of soil nutrients especially phosphorus and iron that are very important nutrients for plant growth and developments (Silva et al., 2023). On the other hand, *Trichoderma* spp. are also used as biofertilizers due to its nitrogen fixing ability in the root zone that also promote the growth of chickpea and increase the yield of crop (Bononi et al., 2020). Moreover, it can make protective layer around the roots and produce plant growth promoting substances like auxins and gibberellins that proliferate the root system and increase the root surface area of chickpea that ultimately leads to availability of more water and nutrients and these resources also trigger the plant growth (Song et al., 2023).

In the present study, the consortia of three *Trichoderma* species was found more effective in alleviating the bad impacts of FOC, thereby enhancing the growth of chickpea. Moreover, these enhancement effects were found dose dependent. Comparable results have been reported in previous studies (Kumari et al., 2020; Worlu et al., 2023).

The variations recorded in the DI, DS and growth of chickpea plants during two growing seasons can be attributed to variations in the environmental factors that greatly influenced the infection levels of FOC on one hand while, affecting the plant growth on the other hand, as discussed in earlier investigations. High moisture contents tend to decrease the *Fusarium* infection in crops (Shabani & Kumar, 2013). Chakrapani et al. (2023) reported 23–27 °C temperature is favorable for the growth of *Fusarium.* In another study, in the *Fusarium* spp. control treatment, DI was low (7.8%) at 22 °C, severe (77% to 82.5%) at 27 °C, and moderate (45%) at 32 °C (Larkin & Fravel, 2002). Stover (1953) reported that *Fusarium* show optimum growth and survival at 15% saturation as *Fusarium* is aerobic and its growth can be greatly reduced by maintaining soil in saturated conditions. In another study, 40%, 50%, and 60% soil moisture showed maximum (66.7%, 58.3%, and 50%) reduction in disease incidence in bell pepper (Attri, Sharma & Gupta, 2018). Variations recorded in the disease suppression in the present study can be attributed to changes in the weather conditions during the critical stage of chickpea plants where high rain fall during the month of January 2022 benefitted *Trichoderma* and chickpea plants on one hand while, it resulted in less disease due to inability of *Fusarium* to survive at higher moisture.

In the present study, soil used in pot experiments was sandy loam. This soil texture corresponds to soil texture of chickpea growing fields to isolate *Fusarium* and indigenous *Trichoderma* spp. Sandy loam soil texture influences the optimum growth of *Fusarium oxysporum* f. sp. *ciceri* (Mergewar et al., 2023). Similarly, the soil pH = 7 influences the optimum growth of *F. oxysporum* f. sp. *ciceri* (Pempee et al., 2020). Soil macro and micronutrients also influence the *Fusarium* wilt in chickpea. High level of nitrogen increases the wilt incidence. On the other hand, increase in soil phosphorus tends to decrease the wilt incidence. Although, in year 2 the concentration of phosphorus was significantly low, but this difference may not affect the disease in chickpea because the impact of environmental factors on disease cannot be ignored. Similarly, zinc is found to be the most effective micronutrient in controlling the wilt incidence (Nathawat et al., 2024). In the present study, the concentration of Zn in both years of pot experiments was found non significant, so the level of disease on chickpea plants cannot be attributed to Zn concentration. Low iron concentration tends to decrease the *Fusarium* wilt disease as *Pseudomonas* spp. present in the soil produce more salicylic acid under low iron concentration and this salicylic acid is responsible for the development of resistance against *F. oxysporum* f. sp. *ciceri* in the host plant as compared to conditions under high iron content in soil where less salicylic acid is produced (Saikia et al., 2005). Although the concentration of Fe in year 1 was significantly low as compared to in year 2 but this decreased Fe concentration cannot be viewed as having impact on lower disease observed in year 2 as the soil was sterilized in pots. On the contrary, high copper content in soil is considered beneficial as it induces resistance in plants against wilt incidence (Graham & Webb, 1991). In our study, the significantly higher concentration of Cu in year 2 may be the cause of low disease on chickpea in year 2, but how much concentrations of macro and micronutrients are required to influence chickpea Fusarium wilt disease need to be investigated in further studies.

As the consortia of *Trichoderma* spp. significantly reduced the *Fusarium* wilt in chickpeas, so, based on these results, it is strongly recommended that the secondary metabolites that are produced and showed mycoparasitism should be isolated through bioguided bioassays. Moreover, the effects of *Trichoderma* species either singly or in the form of consortia, need to be investigated for their effects on plant growth in the absence of pathogen. Similarly, the effects of *Trichoderma* as well as *F. oxysporum* in chickpea and other crops need to be investigated on the soil properties and nutritional characteristics, not covered in the present study.

## Conclusions

In the present study, formulations containing *T. asperellum*, *T. harzianum* strain 1, and *T. harzianum* strain 2, either singly or in the form of consortia, effectively controlled the *Fusarium* wilt disease in chickpea in both years of outdoor pot experiments. However, the formulation comprising the combination of these *Trichoderma* species in the form of consortia was found more effective in controlling the wilt disease in chickpea. This treatment also enhanced the morphological growth of chickpea plants and increased the shoot length, shoot fresh, and dry weight of chickpea plants during the years 1 and 2, respectively, in comparison with infested control plants. Although *Trichoderma* spp. exhibited consistency in controlling the *Fusarium* infection in chickpea plants, the variations of weather during the susceptible stage of chickpea plants influenced the disease levels as well as plant growth. The findings of present study suggest the use of *Trichoderma* spp. to manage the *Fusarium* wilt of chickpeas.

## Supplemental Information

10.7717/peerj.17835/supp-1 Supplemental Information 1 Author photo gel picture, Fasta Sequence *Fusarium oxysporum* f. sp. *ciceris* & Sequences for NCBI *Bankit* The gel picture for gel run, Fasta Sequence *Fusarium oxysporum* f. sp. *ciceris* and sequences for NCBI Bankit. Photo Credit: Safeer Akbar Chohan

10.7717/peerj.17835/supp-2 Supplemental Information 2 Raw values for statistical analysis and Minitab results In pot experiments, Year 1 & 2, all values of disease related parameters and morphological parameters.

10.7717/peerj.17835/supp-3 Supplemental Information 3 Soil analyses All Minitab results are given.

10.7717/peerj.17835/supp-4 Supplemental Information 4 Experiment picture showing the effects of treatments on chickpea Photo Credit: Safeer Akbar Chohan.

## Additional Information and Declarations

### Field Study Permissions

The following information was supplied relating to field study approvals (i.e., approving body and any reference numbers): Field related experiments were approved by Crop Diseases Research Institute (CDRI), National Agricultural Research Centre, Islamabad, vide Dy No. 10712.

### DNA Deposition

The following information was supplied regarding the deposition of DNA sequences: The sequences described are available at GenBank: *Trichoderma asperellum*, Isolate W116499, ON862745; *Trichoderma harzianum* Isolate SA623043, ON862746; *Trichoderma harzianum* Isolate SA1522759 ON862747; *Fusarium oxysporum* f. sp. *ciceris*, Isolate W04FOC, OR808009.

### Data Availability

The following information was supplied regarding data availability: The raw data and the raw measurements are available in the Supplemental File.

## References

[ref-1] Al-Surhanee AA (2022). Protective role of antifusarial eco-friendly agents (*Trichoderma* and salicylic acid) to improve resistance performance of tomato plants. Saudi Journal of Biological Sciences.

[ref-2] Ali B, Terefe H (2021). Spatial distribution and characterization of *Fusarium* wilt (*Fusarium oxysporum* f. sp. *ciceris*) of chickpea in Northern Shoa, Ethiopia. Archives of Phytopathology and Plant Protection.

[ref-3] Anwar R, Ali S, Zeshan MA, Ghani MU, Ali A, Iftikhar Y, Bakhsh A (2022). Effect of *Fusarium* wilt disease on varied chickpea germplasm and its management. Journal of Pure and Applied Agriculture.

[ref-4] Attri K, Sharma M, Gupta SK (2018). Influence of edaphic factors on *Fusarium* wilt of bell pepper. International Journal of Bio-resource and Stress Management.

[ref-5] Batta YA (2004). Postharvest biological control of apple gray mold by *Trichoderma harzianum* Rifai formulated in an invert emulsion. Crop Protection.

[ref-6] Bhagat S, Pan S (2011). Evaluation of *Trichoderma* spp. against root rot and wilt of chickpea (*Cicer arietinum* L.). Biopesticides International.

[ref-7] Biswas MK, Ali SJ (2017). Management of *Fusarium* wilt of chickpea (*Cicer arietinum* L.) under the undulating red and lateritic belt of West Bengal. Journal of Mycopathological Research.

[ref-8] Bononi L, Chiaramonte JB, Pansa CC, Moitinho MA, Melo IS (2020). Phosphorus-solubilizing *Trichoderma* spp. from Amazon soils improve soybean plant growth. Scientific Reports.

[ref-9] Brotman Y, Landau U, Cuadros-Inostroza Á, Takayuki T, Fernie AR, Chet I, Viterbo A, Willmitzer L (2013). Correction: *Trichoderma*-plant root colonization: escaping early plant defense responses and activation of the antioxidant machinery for saline stress tolerance. PLOS Pathogens.

[ref-10] Chakrapani K, Chanu WT, Sinha B, Thangjam B, Hasan W, Devi KS, Chakma T, Phurailatpam S, Mishra LK, Singh GM, Khoyumthem P, Saini R (2023). Deciphering growth abilities of *Fusarium oxysporum* f. sp. *pisi* under variable temperature, pH and nitrogen. Frontiers in Microbiology.

[ref-11] Chaverri P, Gazis RO, Samuels GJ (2011). *Trichoderma amazonicum*, a new endophytic species on *Hevea brasiliensis* and *H. guianensis* from the Amazon basin. Mycologia.

[ref-12] De Clercq P, Mason PG, Babendreier D (2011). Benefits and risks of exotic biological control agents. Biocontrol.

[ref-13] Dubey MK, Ubhayasekera W, Sandgren M, Funck Jensen D, Karlsson M (2012). Disruption of the Eng18B ENGase gene in the fungal biocontrol agent *Trichoderma atroviride* affects growth, conidiation and antagonistic ability. PLOS ONE.

[ref-14] Dubey SC, Suresh M, Singh B (2007). Evaluation of *Trichoderma* species against *Fusarium oxysporum* f. sp. *ciceris* for integrated management of chickpea wilt. Biological Control.

[ref-15] Dutta P, Mahanta M, Singh SB, Thakuria D, Deb L, Kumari A, Upamanya GK, Pandey AK (2023). Molecular interaction between plants and *Trichoderma* species against soil-borne plant pathogens. Frontiers in Plant Science.

[ref-16] Estefan G, Sommer R, Ryan J (2013). Methods of soil, plant, and water analysis. A manual for the West Asia and North Africa Region.

[ref-17] Felsenstein J (1985). Confidence limits on phylogenies: an approach using the bootstrap. Evolution.

[ref-18] Gardes M, Bruns TD (1993). ITS primers with enhanced specificity for basidiomycetes-application to the identification of mycorrhizae and rusts. Molecular Ecology.

[ref-19] Ghosh SK, Banerjee S, Sengupta C (2017). Bioassay, characterization and estimation of siderophores from some important antagonistic fungi. Journal of Biopesticides.

[ref-20] Ghosh SK, Pal S (2017). Growth promotion and *Fusarium* wilt disease management ecofriendly in chickpea by *Trichoderma asperellum*. International Journal of Current Research and Academic Review.

[ref-21] Gil SV, Pastor S, March GJ (2009). Quantitative isolation of biocontrol agents *Trichoderma* spp. *Gliocladium*spp. and actinomycetes from soil with culture media. Microbiological Research.

[ref-22] Graham RD, Webb MJ (1991). Micronutrients and disease resistance and tolerance in plants. Micronutrients in Agriculture.

[ref-23] Hashem A, Tabassum B, Abd_Allah EF, Singh B, Singh G, Kumar K, Nayak S, Srinivasa N (2020). Omics Approaches in Chickpea *Fusarium* Wilt Disease Management. Management of fungal pathogens in pulses. Fungal Biology.

[ref-24] Hernández-Melchor DJ, Ferrera-Cerrato R, Alarcón A (2019). *Trichoderma*: agricultural and biotechnological importance, and fermentation systems for producing biomass and enzymes of industrial interest. Chilean Journal of Agricultural & Animal Sciences.

[ref-25] Hoyos-Carvajal L, Orduz S, Bissett J (2009). Growth stimulation in bean (*Phaseolus vulgaris* L.) by *Trichoderma*. Biological Control.

[ref-26] Jamil A, Ashraf S (2020). Utilization of chemical fungicides in managing the wilt disease of chickpea caused by *Fusarium oxysporum* f. sp. *ciceri*. Archives of Phytopathology and Plant Protection.

[ref-27] Jamil FF, Sarwar M, Sarwar N, Khan JA, Zahid MH, Yousaf S, Arshad HMI, Ul Haq I (2010). Genotyping with RAPD markers resolves pathotype diversity in the Ascochyta blight and Fusarium wilt pathogens of chickpea in Pakistan. Pakistan Journal of Botany.

[ref-28] Jendoubi W, Bouhadida M, Boukteb A, Beji M, Kharrat M (2017). *Fusarium* wilt affecting chickpea crop. Agriculture.

[ref-29] Keswani C, Bisen K, Singh V, Sarma BK, Singh HB, Arora N, Mehnaz S, Balestrini R (2016). Formulation technology of biocontrol agents: present status and future prospects. Bioformulations: for Sustainable Agriculture.

[ref-30] Khan MR, Ashraf S, Rasool F, Salati KM, Mohiddin FA, Haque Z (2014). Field performance of *Trichoderma* species against wilt disease complex of chickpea caused by *Fusarium oxysporum* f. sp. *ciceri* and *Rhizoctonia solani*. Turkish Journal of Agriculture and Forestry.

[ref-31] Khan MR, Khan SM, Mohiddin FA (2004). Biological control of *Fusarium* wilt of chickpea through seed treatment with the commercial formulation of *Trichoderma harzianum* and/or *Pseudomonas fluorescens*. Phytopathologia Mediterranea.

[ref-32] Khandelwal M, Datta S, Mehta J, Naruka R, Makhijani K, Sharma G (2012). Isolation, characterization and biomass production of *Trichoderma viride* using various agro products-A biocontrol agent. Advances in Applied Science Research.

[ref-33] Khare E, Kumar S, Kim K (2018). Role of peptaibols and lytic enzymes of *Trichoderma cerinum* Gur1 in biocontrol of *Fusarium oxysporum* and chickpea wilt. Environmental Sustainability.

[ref-34] Kubheka BP, Ziena LW (2022). *Trichoderma*: a biofertilizer and a bio-fungicide for sustainable crop production.

[ref-35] Kumari M, Sharma OP, Bagri RK, Nathawat BDS (2020). Management of wilt disease of lentil through bio control agents and organic amendments in Rajasthan. Journal of Pharmacognosy and Phytochemistry.

[ref-36] Lacap DC, Hyde KD, Liew ECY (2003). An evaluation of the fungal ‘morphotype’ concept based on ribosomal DNA sequences. Fungal Diversity.

[ref-37] Larkin RP, Fravel DR (2002). Effects of varying environmental conditions on biological control of *Fusarium* wilt of tomato by nonpathogenic *Fusarium* spp. Phytopathology.

[ref-38] Martínez-Martínez TO, Guerrero-Aguilar BZ, Pecina-Quintero V, Rivas-Valencia P, González-Pérez E, Angeles-Núñez JG (2020). *Trichoderma harzianum* antagonism against chickpea fusariosis and its biofertilizing effect. Revista Mexicana de Ciencias Agrícolas.

[ref-39] Meher J, Singh SN, Sonkar SS (2018). Growth promotion of chickpea plant on treatment with native isolates of *Trichoderma* spp. Journal of Pharmacognosy and Phytochemistry.

[ref-40] Mergewar AR, Mulekar VG, Sunita JM, Waghmare SV (2023). Effect of various soil types on incidence of *Fusarium oxysporum* f. sp. *ciceri* causing chickpea wilt. The Pharma Innovation.

[ref-41] Mishra RK, Mishra M, Pandey S, Saabale PR (2022). Exploring the indigenous *Trichoderma* strains from pulses rhizosphere and their biocontrol potential against *Fusarium oxysporum* f. sp. *ciceri* in chickpea. Indian Phytopathology.

[ref-42] Mohamed HALA, Haggag WM (2006). Biocontrol potential of salinity tolerant mutants of *Trichoderma harzianum* against *Fusarium oxysporum*. Brazilian Journal of Microbiology.

[ref-43] Muehlbauer FJ, Sarker A, Varshney R, Thudi M, Muehlbauer F (2017). Economic importance of chickpea: production, value, and world trade. The Chickpea Genome. Compendium of plant genomes.

[ref-44] Mukhopadhyay R, Kumar D (2020). *Trichoderma*: a beneficial antifungal agent and insights into its mechanism of biocontrol potential. Egyptian Journal of Biological Pest Control.

[ref-45] Nagamani P, Biswas MK, Bhagat S (2015). Efficacy of native *Trichoderma* spp. for the management of root rot pathogens in chickpea (*Cicer arietinum* L.). Progressive Research –An International Journal.

[ref-46] Nandeesha KL, Huilgol SN (2021). Integrated management of *Fusarium* wilt of chickpea. The Pharma Innovation.

[ref-47] Nathawat BDS, Sharma OP, Kumari M, Shivran H (2024). Effect of nutrients on wilt in chickpea. Legume Research.

[ref-48] Nelissen H, Moloney M, Inzè D (2014). Translational research: from pot to plot. Plant Biotechnology Journal.

[ref-49] Oszust K, Cybulska J, Frąc M (2020). How do *Trichoderma* genus fungi win a nutritional competition battle against soft fruit pathogens? A report on niche overlap nutritional potentiates. International Journal of Molecular Sciences.

[ref-50] Pandey RN, Gohel NM, Jaisani P (2017). Management of wilt and root rot of chickpea caused by *Fusarium oxysporum* f. sp. *ciceri* and *Macrophomina phaseolina* through seed biopriming and soil application of bio-agents. International Journal of Current Microbiology and Applied Sciences.

[ref-51] Pastor N, Palacios S, Torres AM (2023). Microbial consortia containing fungal biocontrol agents, with emphasis on *Trichoderma* spp.: current applications for plant protection and effects on soil microbial communities. European Journal of Plant Pathology.

[ref-52] Singh M, Prajapati S, Kumari P, Pempee (2020). Effects of different temperature, pH and relative humidity on the growth of *Fusarium oxysporum* f. sp. *ciceri* causing chickpea wilt. International Journal of Current Microbiology and Applied Sciences.

[ref-53] Ponsankar A, Senthil-Nathan S, Vasantha-Srinivasan P, Pandiyan R, Karthi S, Kalaivani K, Chellappandian M, Narayanaswamy R, Thanigaivel A, Patcharin K, Mahboob S, Al-Ghanim KA (2023). Systematic induced resistance in *Solanum lycopersicum* (L.) against vascular wilt pathogen (*Fusarium oxysporum* f. sp. *lycopersici*) by *Citrullus colocynthis* and *Trichoderma viride*. PLOS ONE.

[ref-54] Poromarto SH, Permatasari FI (2023). Fungicide resistance of *Fusarium oxysporum* f. sp. *cepae* isolated from shallot in Brebes. IOP Conference Series: Earth and Environmental Science.

[ref-55] Queirozazevedo DM, Da Silva Rocha F, Fernandes MDFG, Da Costa CA, Muniz MDFS, Barroso PD, Do Rosário Barbosa DMC (2020). Antagonistic effect of *Trichoderma* isolates and its metabolites against *Fusarium solani* and *F. oxysporum* in chickpea. Brazilian Journal of Development.

[ref-56] Ramanagouda G, Naik MK, Sharma M (2022). Biocontrol potentials of novel indigenous *Trichoderma* isolates against *Fusarium* wilt of chickpea. Indian Phytopathology.

[ref-57] Rashid A, Ishaque M, Hameed K, Shabbir M, Ahmad M (2013). Growth and yield response of three chickpea cultivars to varying NPK levels. Asian Journal of Agriculture and Biology.

[ref-58] Rocha FS, Sharma M, Tarafdar A, Chen W, Azevedo DM, Castillo P, Costa CA, Chobe DR (2023). Diseases of Chickpea. Handbook of vegetable and herb diseases.

[ref-59] Roorkiwal M, Jain A, Kale SM, Doddamani D, Chitikineni A, Thudi M (2018). Development and evaluation of high-density Axiom^^®^^*CicerSNP* array for high-resolution genetic mapping and breeding applications in chickpea. Plant Biotechnology Journal.

[ref-60] Ru Z, Di W (2012). *Trichoderma* spp. from rhizosphere soil and their antagonism against *Fusarium sambucinum*. African Journal of Biotechnology.

[ref-61] Rudresh DL, Shivaprakash MK, Prasad RD (2005). Effect of combined application of *Rhizobium*, phosphate solubilizing bacterium and *Trichoderma* spp. on growth, nutrient uptake and yield of chickpea (*Cicer aritenium* L). Applied Soil Ecology.

[ref-62] Ruiz-Cisneros MF, Ornelas-Paz JDJ, Olivas-Orozco GI, Acosta-Muñiz CH, Sepúlveda-Ahumada DR, Pérez-Corral DA, Velasco CR, Salas-Marina MA, Fernández-Pavía SP (2018). Effect of *Trichoderma* spp. and phytopathogenic fungi on plant growth and tomato fruit quality. Revista Mexicana de Fitopatología.

[ref-63] Saikia R, Srivastava AK, Singh K, Arora DK, Lee MW (2005). Effect of iron availability on induction of systemic resistance to *Fusarium* wilt of chickpea by *Pseudomonas* spp. Mycobiology.

[ref-64] Saitou N, Nei M (1987). The neighbor-joining method: a new method for reconstructing phylogenetic trees. Molecular Biology and Evolution.

[ref-65] Sánchez-Montesinos B, Santos M, Moreno-Gavíra A, Marín-Rodulfo T, Gea FJ, Diánez F (2021). Biological control of fungal diseases by *Trichoderma aggressivum* f. *europaeum* and its compatibility with fungicides. Journal of Fungi.

[ref-66] Segaran G, Shankar S, Sathiavelu M (2022). Biocontrol mechanisms of endophytic microorganisms.

[ref-67] Shabani F, Kumar L (2013). Risk levels of invasive *Fusarium oxysporum* f. sp. in areas suitable for date palm (*Phoenix dactylifera*) cultivation under various climate change projections. PLOS ONE.

[ref-68] Shanmugam V, Chugh P, Sharma P (2015). Cold-tolerant *Trichoderma* species for the management of *Fusarium* wilt of tomato plants. Annals of Microbiology.

[ref-69] Sharma IP, Sharma AK (2020). *Trichoderma–Fusarium* interactions: a biocontrol strategy to manage wilt. *Trichoderma*.

[ref-70] Siddiqui ZA, Singh LP (2004). Effects of soil inoculants on the growth, transpiration and wilt disease of chickpea. Journal of Plant Diseases and Protection.

[ref-71] Silva LID, Pereira MC, Carvalho AMXD, Buttrós VH, Pasqual M, Dória J (2023). Phosphorus-solubilizing microorganisms: a key to sustainable agriculture. Agriculture.

[ref-72] Singh SP, Singh HB, Singh DK, Rakshit A (2014). *Trichoderma*-mediated enhancement of nutrient uptake and reduction in incidence of *Rhizoctonia solani* in tomato. Egyptian Journal of Biology.

[ref-73] Singh C, Vyas D (2023). Use of *Ganoderma lucidum* extract to elevate the resistance in chickpea against the *Fusarium oxysporum* f. sp. *ciceris*. Archives of Phytopathology and Plant Protection.

[ref-74] Singh SS, Yadav SK (2007). Comparative efficacy of insecticides, biopesticides and neem formulations against *Helicoverpa armigera* on chickpea. Annals of Plant Protection Sciences.

[ref-75] Song M, Wang X, Xu H, Zhou X, Mu C (2023). Effect of *Trichoderma viride* on insoluble phosphorus absorption ability and growth of *Melilotus officinalis*. Scientific Reports.

[ref-76] Stover RH (1953). The effect of soil moisture on *Fusarium* species. Canadian Journal of Botany.

[ref-77] Tamura K, Nei M, Kumar S (2004). Prospects for inferring very large phylogenies by using the neighbor-joining method. Proceedings of the National Academy of Sciences of the United States of America.

[ref-78] Tamura K, Stecher G, Kumar S (2021). MEGA 11: molecular evolutionary genetics analysis version 11. Molecular Biology and Evolution.

[ref-79] Tomar P, Thakur N, Yadav AN (2022). Endosymbiotic microbes from entomopathogenic nematode (EPNs) and their applications as biocontrol agents for agro-environmental sustainability. Egyptian Journal of Biological Pest Control.

[ref-80] Trivedi S, Srivastava M, Ratan V, Mishra A, Dixit S, Pandey S (2020). Evaluation of microbial consortia on systemic resistance against chickpea wilt. Bangladesh Journal of Botany.

[ref-81] Turkan S, Mierek-Adamska A, Kulasek M, Konieczna WB, Dąbrowska GB (2023). New seed coating containing *Trichoderma viride* with anti-pathogenic properties. PeerJ.

[ref-82] Tyśkiewicz R, Nowak A, Ozimek E, Jaroszuk-Ściseł J (2022). *Trichoderma*: The current status of its application in agriculture for the biocontrol of fungal phytopathogens and stimulation of plant growth. International Journal of Molecular Sciences.

[ref-83] Watanabe T (2010). Pictorial atlas of soil and seed fungi: morphologies of cultured fungi and key to species.

[ref-84] White TJ, Bruns T, Lee SJWT, Taylor J (1990). Amplification and direct sequencing of fungal ribosomal RNA genes for phylogenetics. PCR protocols, A guide to methods and applications.

[ref-85] Wonglom P, Daengsuwan W, Ito SI, Sunpapao A (2019). Biological control of *Sclerotium* fruit rot of snake fruit and stem rot of lettuce by *Trichoderma* sp. T76-12/2 and the mechanisms involved. Physiological and Molecular Plant Pathology.

[ref-86] Woo SL, Scala F, Ruocco M, Lorito M (2006). The molecular biology of the interactions between *Trichoderma* spp. phytopathogenic fungi, and plants. Phytopathology.

[ref-87] Worlu CW, Okogbule FNC, Kpekot KA, Worlu AA (2023). Examination of solubilizing effects of *Trichoderma koningii* and *Trichoderma harzianum* and impact on growth parameters and yield of *Zea mays*. Journal of Agriculture, Environmental Resources and Management.

[ref-88] Xu S, Wang J, Wang H, Bao Y, Li Y, Govindaraju M, Yao W, Chen B, Zhang M (2019). Molecular characterization of carbendazim resistance of *Fusarium* species complex that causes sugarcane pokkah boeng disease. BMC Genomics.

[ref-89] Yazid SNE, Tajudin NI, Razman NAA, Selamat J, Ismail SI, Sanny M, Samsudin NIP (2023). Mycotoxigenic fungal growth inhibition and multi-mycotoxin reduction of potential biological control agents indigenous to grain maize. Mycotoxin Research.

[ref-90] Yedidia I, Srivastva AK, Kapulnik Y, Chet I (2001). Effect of *Trichoderma harzianum* on microelement concentrations and increased growth of cucumber plants. Plant and Soil.

[ref-91] Younesi H, Bazgir E, Darvishnia M, Chehri K (2021). Selection and control efficiency of *Trichoderma* isolates against *Fusarium oxysporum* f. sp. *ciceris* in Iran. Physiological and Molecular Plant Pathology.

[ref-92] Zhang H, Song J, Zhang Z, Zhang Q, Chen S, Mei J, Yu Y, Fang H (2021). Exposure to fungicide difenoconazole reduces the soil bacterial community diversity and the co-occurrence network complexity. Journal of Hazardous Materials.

