# Supplementary figures and images for: Trichoderma based formulations control the wilt disease of chickpea (Cicer arietinum L.) caused by Fusarium oxysporum f. sp. ciceris, better when inoculated as consortia: findings from pot experiments under field conditions
Source: PeerJ. 2024 Aug 19;12:e17835. doi: 10.7717/peerj.17835 (PMC11340631; doi:10.7717/peerj.17835)

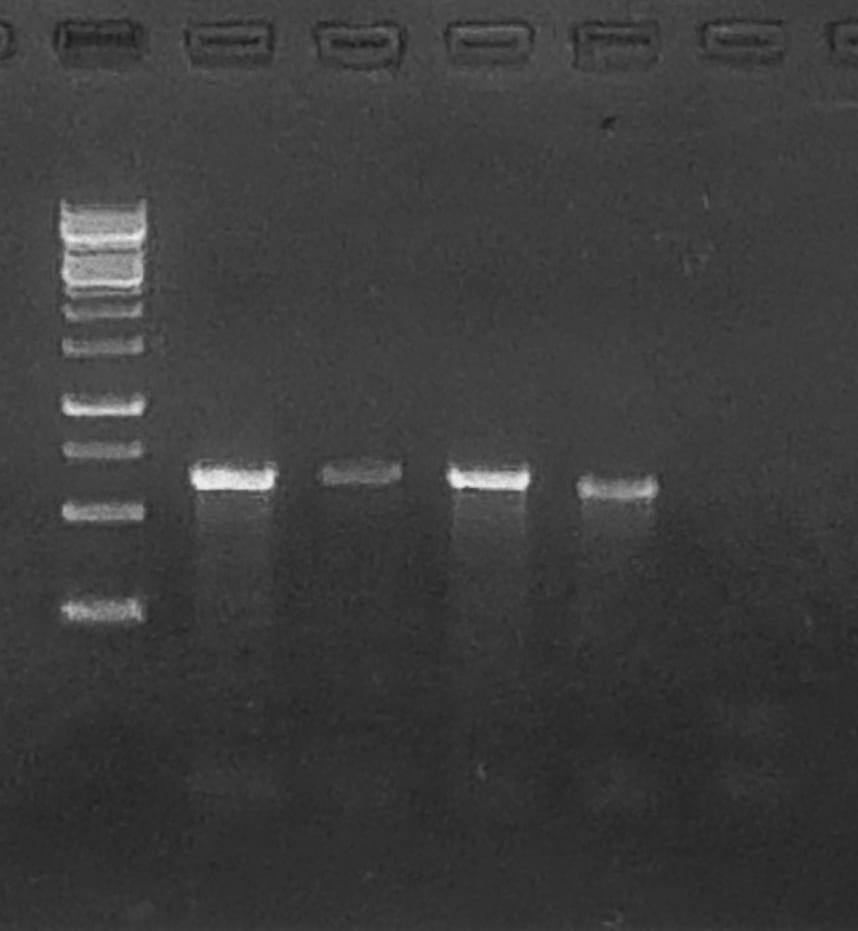

Supplement: Supplemental Information 1 — The gel picture for gel run, Fasta Sequence Fusarium oxysporum f. sp. ciceris and sequences for NCBI Bankit. Photo Credit: Safeer Akbar Chohan [file peerj-12-17835-s001.zip › supplementary file 1/Author photo gel pic.jpg]

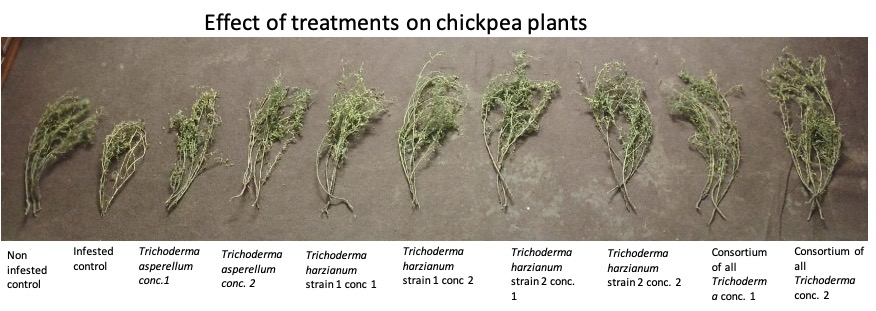

Supplement: Supplemental Information 4 — Photo Credit: Safeer Akbar Chohan. [file peerj-12-17835-s004.jpg]
