# Supplementary material for: Trichoderma based formulations control the wilt disease of chickpea (Cicer arietinum L.) caused by Fusarium oxysporum f. sp. ciceris, better when inoculated as consortia: findings from pot experiments under field conditions
Source: PeerJ. 2024 Aug 19;12:e17835. doi: 10.7717/peerj.17835 (PMC11340631; doi:10.7717/peerj.17835)
Supplement: Supplemental Information 2 — In pot experiments, Year 1 & 2, all values of disease related parameters and morphological parameters. [file peerj-12-17835-s002.zip › supplementary file 2/ANOVA file.docx]

**Dual Culture Length ANOVA**

**Analysis of Variance**

| **Source** | **DF** | **Adj SS** | **Adj MS** | **F-Value** | **P-Value** |
| --- | --- | --- | --- | --- | --- |
| Treatments | 3 | 10.822 | 3.6075 | 11.54 | 0.003 |
| Error | 8 | 2.500 | 0.3125 |  |  |
| Total | 11 | 13.322 |  |  |  |

**Fisher Pairwise Comparisons**

**Grouping Information Using the Fisher LSD Method and 95% Confidence**

| **Treatments** | **N** | **Mean** | **Grouping** | |
| --- | --- | --- | --- | --- |
| Fusarium 114 | 3 | 4.400 | A |  |
| T 538 | 3 | 2.400 |  | B |
| T 130 | 3 | 2.300 |  | B |
| T 507 | 3 | 2.000 |  | B |

*Means that do not share a letter are significantly different.*

**Dual Culture Width ANOVA**

**Analysis of Variance**

| **Source** | **DF** | **Adj SS** | **Adj MS** | **F-Value** | **P-Value** |
| --- | --- | --- | --- | --- | --- |
| Treatments | 3 | 11.130 | 3.7100 | 12.16 | 0.002 |
| Error | 8 | 2.440 | 0.3050 |  |  |
| Total | 11 | 13.570 |  |  |  |

**Fisher Pairwise Comparisons**

**Grouping Information Using the Fisher LSD Method and 95% Confidence**

| **Treatments** | **N** | **Mean** | **Grouping** | |
| --- | --- | --- | --- | --- |
| Fusarium 114 | 3 | 6.500 | A |  |
| T 130 | 3 | 4.500 |  | B |
| T 507 | 3 | 4.300 |  | B |
| T 538 | 3 | 4.100 |  | B |

*Means that do not share a letter are significantly different.*

**Dual Culture Average ANOVA**

**Analysis of Variance**

| **Source** | **DF** | **Adj SS** | **Adj MS** | **F-Value** | **P-Value** |
| --- | --- | --- | --- | --- | --- |
| Treatments | 3 | 10.821 | 3.6069 | 19.30 | 0.001 |
| Error | 8 | 1.495 | 0.1869 |  |  |
| Total | 11 | 12.316 |  |  |  |

**Fisher Pairwise Comparisons**

**Grouping Information Using the Fisher LSD Method and 95% Confidence**

| **Treatments** | **N** | **Mean** | **Grouping** | |
| --- | --- | --- | --- | --- |
| Fusarium 114 | 3 | 5.450 | A |  |
| T 130 | 3 | 3.400 |  | B |
| T 538 | 3 | 3.250 |  | B |
| T 507 | 3 | 3.150 |  | B |

*Means that do not share a letter are significantly different.*

**Year 1**

**Shoot Length**

**Analysis of Variance**

| **Source** | **DF** | **Adj SS** | **Adj MS** | **F-Value** | **P-Value** |
| --- | --- | --- | --- | --- | --- |
| Treatment | 9 | 480.48 | 53.387 | 17.97 | 0.000 |
| Error | 20 | 59.42 | 2.971 |  |  |
| Total | 29 | 539.90 |  |  |  |

**Fisher Pairwise Comparisons**

**Grouping Information Using the Fisher LSD Method and 95% Confidence**

| **Treatment** | **N** | **Mean** | **Grouping** | | | | | |
| --- | --- | --- | --- | --- | --- | --- | --- | --- |
| Cons conc.2 | 3 | 38.000 | A |  |  |  |  |  |
| Cons conc.1 | 3 | 35.000 |  | B |  |  |  |  |
| T.h S2 conc.2 | 3 | 32.800 |  | B | C |  |  |  |
| T.h S2 conc.1 | 3 | 31.300 |  |  | C | D |  |  |
| T.h S1 conc.2 | 3 | 31.200 |  |  | C | D |  |  |
| NC | 3 | 31.000 |  |  | C | D |  |  |
| T.h S1 conc.1 | 3 | 29.700 |  |  |  | D | E |  |
| T.a conc.2 | 3 | 29.50 |  |  |  | D | E |  |
| T.a conc.1 | 3 | 27.00 |  |  |  |  | E |  |
| PC | 3 | 22.50 |  |  |  |  |  | F |

*Means that do not share a letter are significantly different.*

Shoot Fresh Weight Year 1

**Analysis of Variance**

| **Source** | **DF** | **Adj SS** | **Adj MS** | **F-Value** | **P-Value** |
| --- | --- | --- | --- | --- | --- |
| Treatment | 9 | 103.05 | 11.450 | 10.73 | 0.000 |
| Error | 20 | 21.34 | 1.067 |  |  |
| Total | 29 | 124.39 |  |  |  |

**Fisher Pairwise Comparisons**

**Grouping Information Using the Fisher LSD Method and 95% Confidence**

| **Treatment** | **N** | **Mean** | **Grouping** | | | | |
| --- | --- | --- | --- | --- | --- | --- | --- |
| NC | 3 | 15.300 | A |  |  |  |  |
| Cons conc.2 | 3 | 14.500 | A |  |  |  |  |
| Cons conc.1 | 3 | 13.900 | A | B |  |  |  |
| T.h S2 conc.2 | 3 | 12.500 |  | B | C |  |  |
| T.h S2 conc.1 | 3 | 12.000 |  |  | C | D |  |
| T.h S1 conc.2 | 3 | 11.900 |  |  | C | D |  |
| T.a conc.2 | 3 | 11.450 |  |  | C | D |  |
| T.h S1 conc.1 | 3 | 11.400 |  |  | C | D |  |
| T.a conc.1 | 3 | 10.500 |  |  |  | D |  |
| PC | 3 | 8.700 |  |  |  |  | E |

*Means that do not share a letter are significantly different.*

Shoot Dry weight Year 1

**Analysis of Variance**

| **Source** | **DF** | **Adj SS** | **Adj MS** | **F-Value** | **P-Value** |
| --- | --- | --- | --- | --- | --- |
| Treatment | 9 | 30.075 | 3.3417 | 12.36 | 0.000 |
| Error | 20 | 5.407 | 0.2703 |  |  |
| Total | 29 | 35.482 |  |  |  |

**Fisher Pairwise Comparisons**

**Grouping Information Using the Fisher LSD Method and 95% Confidence**

| **Treatment** | **N** | **Mean** | **Grouping** | | | | |
| --- | --- | --- | --- | --- | --- | --- | --- |
| NC | 3 | 8.500 | A |  |  |  |  |
| Cons conc.2 | 3 | 8.400 | A |  |  |  |  |
| Cons conc.1 | 3 | 7.800 | A | B |  |  |  |
| T.h S2 conc.2 | 3 | 7.100 |  | B | C |  |  |
| T.h S1 conc.2 | 3 | 6.900 |  |  | C | D |  |
| T.h S2 conc.1 | 3 | 6.833 |  |  | C | D |  |
| T.h S1 conc.1 | 3 | 6.600 |  |  | C | D |  |
| T.a conc.2 | 3 | 6.600 |  |  | C | D |  |
| T.a conc.1 | 3 | 6.100 |  |  |  | D |  |
| PC | 3 | 5.000 |  |  |  |  | E |

*Means that do not share a letter are significantly different.*

Disease Severity Year 1

**Analysis of Variance**

| **Source** | **DF** | **Adj SS** | **Adj MS** | **F-Value** | **P-Value** |
| --- | --- | --- | --- | --- | --- |
| Treatment | 9 | 58.36 | 6.4841 | 12.80 | 0.000 |
| Error | 20 | 10.13 | 0.5066 |  |  |
| Total | 29 | 68.49 |  |  |  |

**Grouping Information Using the Fisher LSD Method and 95% Confidence**

| **Treatment** | **N** | **Mean** | **Grouping** | | | | |
| --- | --- | --- | --- | --- | --- | --- | --- |
| PC | 3 | 4.667 | A |  |  |  |  |
| T.a conc.1 | 3 | 3.677 | A | B |  |  |  |
| T.a conc.2 | 3 | 3.577 | A | B |  |  |  |
| T.h S1 conc.1 | 3 | 3.333 |  | B |  |  |  |
| T.h S1 conc.2 | 3 | 3.233 |  | B |  |  |  |
| T.h S2 conc.1 | 3 | 2.800 |  | B |  |  |  |
| T.h S2 conc.2 | 3 | 2.467 |  | B | C |  |  |
| Cons conc.1 | 3 | 1.343 |  |  | C | D |  |
| Cons conc.2 | 3 | 0.667 |  |  |  | D | E |
| NC | 3 | 0.000000 |  |  |  |  | E |

*Means that do not share a letter are significantly different.*

Disease Incidence Year 1

**Analysis of Variance**

| **Source** | **DF** | **Adj SS** | **Adj MS** | **F-Value** | **P-Value** |
| --- | --- | --- | --- | --- | --- |
| Treatment | 9 | 18.133 | 2.0148 | 4.32 | 0.003 |
| Error | 20 | 9.333 | 0.4667 |  |  |
| Total | 29 | 27.467 |  |  |  |

**Fisher Pairwise Comparisons**

**Grouping Information Using the Fisher LSD Method and 95% Confidence**

| **Treatment** | **N** | **Mean** | **Grouping** | | | | |
| --- | --- | --- | --- | --- | --- | --- | --- |
| PC | 3 | 2.667 | A |  |  |  |  |
| T.a conc.1 | 3 | 2.333 | A | B |  |  |  |
| T.h S1 conc.1 | 3 | 2.000 | A | B | C |  |  |
| T.a conc.2 | 3 | 2.000 | A | B | C |  |  |
| T.h S1 conc.2 | 3 | 1.667 | A | B | C | D |  |
| T.h S2 conc.1 | 3 | 1.333 |  | B | C | D |  |
| T.h S2 conc.2 | 3 | 1.000 |  |  | C | D | E |
| Cons conc.1 | 3 | 1.000 |  |  | C | D | E |
| Cons conc.2 | 3 | 0.667 |  |  |  | D | E |
| NC | 3 | 0.000000 |  |  |  |  | E |

*Means that do not share a letter are significantly different.*

**Shoot length Year 2**

**Analysis of Variance**

| **Source** | **DF** | **Adj SS** | **Adj MS** | **F-Value** | **P-Value** |
| --- | --- | --- | --- | --- | --- |
| Treatment | 9 | 714.15 | 79.350 | 23.16 | 0.000 |
| Error | 20 | 68.52 | 3.426 |  |  |
| Total | 29 | 782.67 |  |  |  |

**Fisher Pairwise Comparisons**

**Grouping Information Using the Fisher LSD Method and 95% Confidence**

| **Treatment** | **N** | **Mean** | **Grouping** | | | | | |
| --- | --- | --- | --- | --- | --- | --- | --- | --- |
| Cons conc.2 | 3 | 43.00 | A |  |  |  |  |  |
| Cons conc.1 | 3 | 41.00 | A | B |  |  |  |  |
| T.h S2 conc.2 | 3 | 38.50 |  | B | C |  |  |  |
| T.h S2 conc.1 | 3 | 37.00 |  |  | C | D |  |  |
| T.h S1 conc.2 | 3 | 36.800 |  |  | C | D |  |  |
| T.h S1 conc.1 | 3 | 35.900 |  |  | C | D |  |  |
| T.a conc.2 | 3 | 34.100 |  |  |  | D | E |  |
| NC | 3 | 32.30 |  |  |  |  | E |  |
| T.a conc.1 | 3 | 31.50 |  |  |  |  | E |  |
| PC | 3 | 25.00 |  |  |  |  |  | F |

*Means that do not share a letter are significantly different.*

Shoot Fresh Weight Year 2

**Analysis of Variance**

| **Source** | **DF** | **Adj SS** | **Adj MS** | **F-Value** | **P-Value** |
| --- | --- | --- | --- | --- | --- |
| Treatment | 9 | 135.03 | 15.003 | 14.99 | 0.000 |
| Error | 20 | 20.02 | 1.001 |  |  |
| Total | 29 | 155.05 |  |  |  |

**Fisher Pairwise Comparisons**

**Grouping Information Using the Fisher LSD Method and 95% Confidence**

| **Treatment** | **N** | **Mean** | **Grouping** | | | | | |
| --- | --- | --- | --- | --- | --- | --- | --- | --- |
| Cons conc.2 | 3 | 18.200 | A |  |  |  |  |  |
| Cons conc.1 | 3 | 17.500 | A | B |  |  |  |  |
| NC | 3 | 16.900 | A | B | C |  |  |  |
| T.h S2 conc.2 | 3 | 16.500 | A | B | C |  |  |  |
| T.h S2 conc.1 | 3 | 15.800 |  | B | C | D |  |  |
| T.h S1 conc.2 | 3 | 15.700 |  |  | C | D |  |  |
| T.h S1 conc.1 | 3 | 15.200 |  |  | C | D |  |  |
| T.a conc.2 | 3 | 14.400 |  |  |  | D | E |  |
| T.a conc.1 | 3 | 13.400 |  |  |  |  | E |  |
| PC | 3 | 10.500 |  |  |  |  |  | F |

*Means that do not share a letter are significantly different.*

Shoot Dry Weight Year 2

**Analysis of Variance**

| **Source** | **DF** | **Adj SS** | **Adj MS** | **F-Value** | **P-Value** |
| --- | --- | --- | --- | --- | --- |
| Treatment | 9 | 46.128 | 5.1253 | 14.94 | 0.000 |
| Error | 20 | 6.860 | 0.3430 |  |  |
| Total | 29 | 52.988 |  |  |  |

**Fisher Pairwise Comparisons**

**Grouping Information Using the Fisher LSD Method and 95% Confidence**

| **Treatment** | **N** | **Mean** | **Grouping** | | | | | | |
| --- | --- | --- | --- | --- | --- | --- | --- | --- | --- |
| Cons conc.2 | 3 | 10.300 | A |  |  |  |  |  |  |
| Cons conc.1 | 3 | 9.900 | A | B |  |  |  |  |  |
| T.h S2 conc.2 | 3 | 9.600 | A | B | C |  |  |  |  |
| T.h S2 conc.1 | 3 | 9.200 |  | B | C | D |  |  |  |
| NC | 3 | 9.200 |  | B | C | D |  |  |  |
| T.h S1 conc.2 | 3 | 8.800 |  |  | C | D | E |  |  |
| T.h S1 conc.1 | 3 | 8.500 |  |  |  | D | E |  |  |
| T.a conc.2 | 3 | 8.000 |  |  |  |  | E | F |  |
| T.a conc.1 | 3 | 7.400 |  |  |  |  |  | F |  |
| PC | 3 | 5.900 |  |  |  |  |  |  | G |

*Means that do not share a letter are significantly different.*

Disease Severity ANOVA Year 2

**Analysis of Variance**

| **Source** | **DF** | **Adj SS** | **Adj MS** | **F-Value** | **P-Value** |
| --- | --- | --- | --- | --- | --- |
| Treatment | 9 | 48.325 | 5.3695 | 15.72 | 0.000 |
| Error | 20 | 6.832 | 0.3416 |  |  |
| Total | 29 | 55.157 |  |  |  |

**Fisher Pairwise Comparisons**

**Grouping Information Using the Fisher LSD Method and 95% Confidence**

| **Treatment** | **N** | **Mean** | **Grouping** | | | | |
| --- | --- | --- | --- | --- | --- | --- | --- |
| PC | 3 | 4.110 | A |  |  |  |  |
| T.a conc.1 | 3 | 3.553 | A | B |  |  |  |
| T.h S1 conc.1 | 3 | 2.887 |  | B |  |  |  |
| T.a conc.2 | 3 | 2.887 |  | B |  |  |  |
| T.h S2 conc.1 | 3 | 2.667 |  | B | C |  |  |
| T.h S1 conc.2 | 3 | 2.667 |  | B | C |  |  |
| T.h S2 conc.2 | 3 | 1.777 |  |  | C | D |  |
| Cons conc.1 | 3 | 1.333 |  |  |  | D |  |
| Cons conc.2 | 3 | 0.333 |  |  |  |  | E |
| NC | 3 | 0.000000 |  |  |  |  | E |

*Means that do not share a letter are significantly different.*

**Disease Incidence Year 2**

**Analysis of Variance**

| **Source** | **DF** | **Adj SS** | **Adj MS** | **F-Value** | **P-Value** |
| --- | --- | --- | --- | --- | --- |
| Treatment | 9 | 14.700 | 1.6333 | 7.00 | 0.000 |
| Error | 20 | 4.667 | 0.2333 |  |  |
| Total | 29 | 19.367 |  |  |  |

**Fisher Pairwise Comparisons**

**Grouping Information Using the Fisher LSD Method and 95% Confidence**

| **Treatment** | **N** | **Mean** | **Grouping** | | | | | |
| --- | --- | --- | --- | --- | --- | --- | --- | --- |
| PC | 3 | 2.333 | A |  |  |  |  |  |
| T.a conc.1 | 3 | 2.000 | A | B |  |  |  |  |
| T.h S1 conc.1 | 3 | 1.667 | A | B | C |  |  |  |
| T.a conc.2 | 3 | 1.667 | A | B | C |  |  |  |
| T.h S2 conc.1 | 3 | 1.333 |  | B | C | D |  |  |
| T.h S1 conc.2 | 3 | 1.333 |  | B | C | D |  |  |
| T.h S2 conc.2 | 3 | 1.000 |  |  | C | D | E |  |
| Cons conc.1 | 3 | 0.667 |  |  |  | D | E | F |
| Cons conc.2 | 3 | 0.333 |  |  |  |  | E | F |
| NC | 3 | 0.000000 |  |  |  |  |  | F |

*Means that do not share a letter are significantly different.*
