# Supplementary material for: Trichoderma based formulations control the wilt disease of chickpea (Cicer arietinum L.) caused by Fusarium oxysporum f. sp. ciceris, better when inoculated as consortia: findings from pot experiments under field conditions
Source: PeerJ. 2024 Aug 19;12:e17835. doi: 10.7717/peerj.17835 (PMC11340631; doi:10.7717/peerj.17835)
Supplement: Supplemental Information 3 — All Minitab results are given. [file peerj-12-17835-s003.zip › supplementary file 3/safeer anova word 26-05-2024.docx]

WORKSHEET 1

**One-way ANOVA: pH versus Treatments**

**Method**

| Null hypothesis | All means are equal |
| --- | --- |
| Alternative hypothesis | Not all means are equal |
| Significance level | α = 0.05 |

*Equal variances were assumed for the analysis.*

**Factor Information**

| **Factor** | **Levels** | **Values** |
| --- | --- | --- |
| Treatments | 4 | c1, c2, T1, T2 |

**Analysis of Variance**

| **Source** | **DF** | **Seq SS** | **Contribution** | **Adj SS** | **Adj MS** | **F-Value** | **P-Value** |
| --- | --- | --- | --- | --- | --- | --- | --- |
| Treatments | 3 | 0.4382 | 56.60% | 0.4382 | 0.14607 | 3.48 | 0.070 |
| Error | 8 | 0.3360 | 43.40% | 0.3360 | 0.04200 |  |  |
| Total | 11 | 0.7742 | 100.00% |  |  |  |  |

**Model Summary**

| **S** | **R-sq** | **R-sq(adj)** | **PRESS** | **R-sq(pred)** |
| --- | --- | --- | --- | --- |
| 0.204939 | 56.60% | 40.33% | 0.756 | 2.35% |

**Means**

| **Treatments** | **N** | **Mean** | **StDev** | **95% CI** |
| --- | --- | --- | --- | --- |
| c1 | 3 | 7.5000 | 0.1400 | (7.2271, 7.7729) |
| c2 | 3 | 7.900 | 0.361 | (7.627, 8.173) |
| T1 | 3 | 7.4000 | 0.1054 | (7.1271, 7.6729) |
| T2 | 3 | 7.5100 | 0.0854 | (7.2371, 7.7829) |

*Pooled StDev = 0.204939*

**Fisher Pairwise Comparisons**

**Grouping Information Using the Fisher LSD Method and 95% Confidence**

| **Treatments** | **N** | **Mean** | **Grouping** | |
| --- | --- | --- | --- | --- |
| c2 | 3 | 7.900 | A |  |
| T2 | 3 | 7.5100 |  | B |
| c1 | 3 | 7.5000 |  | B |
| T1 | 3 | 7.4000 |  | B |

*Means that do not share a letter are significantly different.*

WORKSHEET 1

**One-way ANOVA: SOM versus Treatments**

**Method**

| Null hypothesis | All means are equal |
| --- | --- |
| Alternative hypothesis | Not all means are equal |
| Significance level | α = 0.05 |

*Equal variances were assumed for the analysis.*

**Factor Information**

| **Factor** | **Levels** | **Values** |
| --- | --- | --- |
| Treatments | 4 | c1, c2, T1, T2 |

**Analysis of Variance**

| **Source** | **DF** | **Seq SS** | **Contribution** | **Adj SS** | **Adj MS** | **F-Value** | **P-Value** |
| --- | --- | --- | --- | --- | --- | --- | --- |
| Treatments | 3 | 0.042825 | 87.71% | 0.042825 | 0.014275 | 19.03 | 0.001 |
| Error | 8 | 0.006000 | 12.29% | 0.006000 | 0.000750 |  |  |
| Total | 11 | 0.048825 | 100.00% |  |  |  |  |

**Model Summary**

| **S** | **R-sq** | **R-sq(adj)** | **PRESS** | **R-sq(pred)** |
| --- | --- | --- | --- | --- |
| 0.0273861 | 87.71% | 83.10% | 0.0135 | 72.35% |

**Means**

| **Treatments** | **N** | **Mean** | **StDev** | **95% CI** |
| --- | --- | --- | --- | --- |
| c1 | 3 | 0.4800 | 0.0361 | (0.4435, 0.5165) |
| c2 | 3 | 0.6400 | 0.0265 | (0.6035, 0.6765) |
| T1 | 3 | 0.5500 | 0.0173 | (0.5135, 0.5865) |
| T2 | 3 | 0.6000 | 0.0265 | (0.5635, 0.6365) |

*Pooled StDev = 0.0273861*

**Fisher Pairwise Comparisons**

**Grouping Information Using the Fisher LSD Method and 95% Confidence**

| **Treatments** | **N** | **Mean** | **Grouping** | | |
| --- | --- | --- | --- | --- | --- |
| c2 | 3 | 0.6400 | A |  |  |
| T2 | 3 | 0.6000 | A | B |  |
| T1 | 3 | 0.5500 |  | B |  |
| c1 | 3 | 0.4800 |  |  | C |

*Means that do not share a letter are significantly different.*

WORKSHEET 1

**One-way ANOVA: N versus Treatments**

**Method**

| Null hypothesis | All means are equal |
| --- | --- |
| Alternative hypothesis | Not all means are equal |
| Significance level | α = 0.05 |

*Equal variances were assumed for the analysis.*

**Factor Information**

| **Factor** | **Levels** | **Values** |
| --- | --- | --- |
| Treatments | 4 | c1, c2, T1, T2 |

**Analysis of Variance**

| **Source** | **DF** | **Seq SS** | **Contribution** | **Adj SS** | **Adj MS** | **F-Value** | **P-Value** |
| --- | --- | --- | --- | --- | --- | --- | --- |
| Treatments | 3 | 3.38190 | 98.35% | 3.38190 | 1.12730 | 158.77 | 0.000 |
| Error | 8 | 0.05680 | 1.65% | 0.05680 | 0.00710 |  |  |
| Total | 11 | 3.43870 | 100.00% |  |  |  |  |

**Model Summary**

| **S** | **R-sq** | **R-sq(adj)** | **PRESS** | **R-sq(pred)** |
| --- | --- | --- | --- | --- |
| 0.0842615 | 98.35% | 97.73% | 0.1278 | 96.28% |

**Means**

| **Treatments** | **N** | **Mean** | **StDev** | **95% CI** |
| --- | --- | --- | --- | --- |
| c1 | 3 | 3.5000 | 0.1114 | (3.3878, 3.6122) |
| c2 | 3 | 2.1000 | 0.1000 | (1.9878, 2.2122) |
| T1 | 3 | 2.6500 | 0.0458 | (2.5378, 2.7622) |
| T2 | 3 | 2.3300 | 0.0624 | (2.2178, 2.4422) |

*Pooled StDev = 0.0842615*

**Fisher Pairwise Comparisons**

**Grouping Information Using the Fisher LSD Method and 95% Confidence**

| **Treatments** | **N** | **Mean** | **Grouping** | | | |
| --- | --- | --- | --- | --- | --- | --- |
| c1 | 3 | 3.5000 | A |  |  |  |
| T1 | 3 | 2.6500 |  | B |  |  |
| T2 | 3 | 2.3300 |  |  | C |  |
| c2 | 3 | 2.1000 |  |  |  | D |

*Means that do not share a letter are significantly different.*

WORKSHEET 1

**One-way ANOVA: P versus Treatments**

**Method**

| Null hypothesis | All means are equal |
| --- | --- |
| Alternative hypothesis | Not all means are equal |
| Significance level | α = 0.05 |

*Equal variances were assumed for the analysis.*

**Factor Information**

| **Factor** | **Levels** | **Values** |
| --- | --- | --- |
| Treatments | 4 | c1, c2, T1, T2 |

**Analysis of Variance**

| **Source** | **DF** | **Seq SS** | **Contribution** | **Adj SS** | **Adj MS** | **F-Value** | **P-Value** |
| --- | --- | --- | --- | --- | --- | --- | --- |
| Treatments | 3 | 2.9102 | 83.21% | 2.9102 | 0.97007 | 13.22 | 0.002 |
| Error | 8 | 0.5871 | 16.79% | 0.5871 | 0.07338 |  |  |
| Total | 11 | 3.4973 | 100.00% |  |  |  |  |

**Model Summary**

| **S** | **R-sq** | **R-sq(adj)** | **PRESS** | **R-sq(pred)** |
| --- | --- | --- | --- | --- |
| 0.270894 | 83.21% | 76.92% | 1.3209 | 62.23% |

**Means**

| **Treatments** | **N** | **Mean** | **StDev** | **95% CI** |
| --- | --- | --- | --- | --- |
| c1 | 3 | 5.200 | 0.347 | (4.839, 5.561) |
| c2 | 3 | 6.483 | 0.369 | (6.123, 6.844) |
| T1 | 3 | 6.1400 | 0.1652 | (5.7793, 6.5007) |
| T2 | 3 | 5.6000 | 0.1000 | (5.2393, 5.9607) |

*Pooled StDev = 0.270894*

**Fisher Pairwise Comparisons**

**Grouping Information Using the Fisher LSD Method and 95% Confidence**

| **Treatments** | **N** | **Mean** | **Grouping** | |
| --- | --- | --- | --- | --- |
| c2 | 3 | 6.483 | A |  |
| T1 | 3 | 6.1400 | A |  |
| T2 | 3 | 5.6000 |  | B |
| c1 | 3 | 5.200 |  | B |

*Means that do not share a letter are significantly different.*

wORKSHEET 1

**One-way ANOVA: K versus Treatments**

**Method**

| Null hypothesis | All means are equal |
| --- | --- |
| Alternative hypothesis | Not all means are equal |
| Significance level | α = 0.05 |

*Equal variances were assumed for the analysis.*

**Factor Information**

| **Factor** | **Levels** | **Values** |
| --- | --- | --- |
| Treatments | 4 | c1, c2, T1, T2 |

**Analysis of Variance**

| **Source** | **DF** | **Seq SS** | **Contribution** | **Adj SS** | **Adj MS** | **F-Value** | **P-Value** |
| --- | --- | --- | --- | --- | --- | --- | --- |
| Treatments | 3 | 984.38 | 95.19% | 984.38 | 328.127 | 52.75 | 0.000 |
| Error | 8 | 49.76 | 4.81% | 49.76 | 6.220 |  |  |
| Total | 11 | 1034.14 | 100.00% |  |  |  |  |

**Model Summary**

| **S** | **R-sq** | **R-sq(adj)** | **PRESS** | **R-sq(pred)** |
| --- | --- | --- | --- | --- |
| 2.49400 | 95.19% | 93.38% | 111.961 | 89.17% |

**Means**

| **Treatments** | **N** | **Mean** | **StDev** | **95% CI** |
| --- | --- | --- | --- | --- |
| c1 | 3 | 92.00 | 3.61 | (88.68, 95.32) |
| c2 | 3 | 101.13 | 2.80 | (97.81, 104.45) |
| T1 | 3 | 79.300 | 1.509 | (75.980, 82.620) |
| T2 | 3 | 80.000 | 1.323 | (76.680, 83.320) |

*Pooled StDev = 2.49400*

**Fisher Pairwise Comparisons**

**Grouping Information Using the Fisher LSD Method and 95% Confidence**

| **Treatments** | **N** | **Mean** | **Grouping** | | |
| --- | --- | --- | --- | --- | --- |
| c2 | 3 | 101.13 | A |  |  |
| c1 | 3 | 92.00 |  | B |  |
| T2 | 3 | 80.000 |  |  | C |
| T1 | 3 | 79.300 |  |  | C |

*Means that do not share a letter are significantly different.*

WORKSHEET 1

**One-way ANOVA: Zn versus Treatments**

**Method**

| Null hypothesis | All means are equal |
| --- | --- |
| Alternative hypothesis | Not all means are equal |
| Significance level | α = 0.05 |

*Equal variances were assumed for the analysis.*

**Factor Information**

| **Factor** | **Levels** | **Values** |
| --- | --- | --- |
| Treatments | 4 | c1, c2, T1, T2 |

**Analysis of Variance**

| **Source** | **DF** | **Seq SS** | **Contribution** | **Adj SS** | **Adj MS** | **F-Value** | **P-Value** |
| --- | --- | --- | --- | --- | --- | --- | --- |
| Treatments | 3 | 0.24743 | 94.57% | 0.24743 | 0.082475 | 46.46 | 0.000 |
| Error | 8 | 0.01420 | 5.43% | 0.01420 | 0.001775 |  |  |
| Total | 11 | 0.26163 | 100.00% |  |  |  |  |

**Model Summary**

| **S** | **R-sq** | **R-sq(adj)** | **PRESS** | **R-sq(pred)** |
| --- | --- | --- | --- | --- |
| 0.0421307 | 94.57% | 92.54% | 0.03195 | 87.79% |

**Means**

| **Treatments** | **N** | **Mean** | **StDev** | **95% CI** |
| --- | --- | --- | --- | --- |
| c1 | 3 | 0.9100 | 0.0346 | (0.8539, 0.9661) |
| c2 | 3 | 0.9400 | 0.0700 | (0.8839, 0.9961) |
| T1 | 3 | 0.6600 | 0.0265 | (0.6039, 0.7161) |
| T2 | 3 | 0.6200 | 0.0173 | (0.5639, 0.6761) |

*Pooled StDev = 0.0421307*

**Fisher Pairwise Comparisons**

**Grouping Information Using the Fisher LSD Method and 95% Confidence**

| **Treatments** | **N** | **Mean** | **Grouping** | |
| --- | --- | --- | --- | --- |
| c2 | 3 | 0.9400 | A |  |
| c1 | 3 | 0.9100 | A |  |
| T1 | 3 | 0.6600 |  | B |
| T2 | 3 | 0.6200 |  | B |

*Means that do not share a letter are significantly different.*

WORKSHEET 1

**One-way ANOVA: Fe versus Treatments**

**Method**

| Null hypothesis | All means are equal |
| --- | --- |
| Alternative hypothesis | Not all means are equal |
| Significance level | α = 0.05 |

*Equal variances were assumed for the analysis.*

**Factor Information**

| **Factor** | **Levels** | **Values** |
| --- | --- | --- |
| Treatments | 4 | c1, c2, T1, T2 |

**Analysis of Variance**

| **Source** | **DF** | **Seq SS** | **Contribution** | **Adj SS** | **Adj MS** | **F-Value** | **P-Value** |
| --- | --- | --- | --- | --- | --- | --- | --- |
| Treatments | 3 | 44.356 | 97.18% | 44.356 | 14.7855 | 91.80 | 0.000 |
| Error | 8 | 1.288 | 2.82% | 1.288 | 0.1611 |  |  |
| Total | 11 | 45.645 | 100.00% |  |  |  |  |

**Model Summary**

| **S** | **R-sq** | **R-sq(adj)** | **PRESS** | **R-sq(pred)** |
| --- | --- | --- | --- | --- |
| 0.401321 | 97.18% | 96.12% | 2.89905 | 93.65% |

**Means**

| **Treatments** | **N** | **Mean** | **StDev** | **95% CI** |
| --- | --- | --- | --- | --- |
| c1 | 3 | 6.030 | 0.197 | (5.496, 6.564) |
| c2 | 3 | 5.0033 | 0.0896 | (4.4690, 5.5376) |
| T1 | 3 | 9.700 | 0.709 | (9.166, 10.234) |
| T2 | 3 | 8.770 | 0.308 | (8.236, 9.304) |

*Pooled StDev = 0.401321*

**Fisher Pairwise Comparisons**

**Grouping Information Using the Fisher LSD Method and 95% Confidence**

| **Treatments** | **N** | **Mean** | **Grouping** | | | |
| --- | --- | --- | --- | --- | --- | --- |
| T1 | 3 | 9.700 | A |  |  |  |
| T2 | 3 | 8.770 |  | B |  |  |
| c1 | 3 | 6.030 |  |  | C |  |
| c2 | 3 | 5.0033 |  |  |  | D |

*Means that do not share a letter are significantly different.*

wORKSHEET 1

**One-way ANOVA: Cu versus Treatments**

**Method**

| Null hypothesis | All means are equal |
| --- | --- |
| Alternative hypothesis | Not all means are equal |
| Significance level | α = 0.05 |

*Equal variances were assumed for the analysis.*

**Factor Information**

| **Factor** | **Levels** | **Values** |
| --- | --- | --- |
| Treatments | 4 | c1, c2, T1, T2 |

**Analysis of Variance**

| **Source** | **DF** | **Seq SS** | **Contribution** | **Adj SS** | **Adj MS** | **F-Value** | **P-Value** |
| --- | --- | --- | --- | --- | --- | --- | --- |
| Treatments | 3 | 1.58962 | 99.31% | 1.58962 | 0.529875 | 385.36 | 0.000 |
| Error | 8 | 0.01100 | 0.69% | 0.01100 | 0.001375 |  |  |
| Total | 11 | 1.60062 | 100.00% |  |  |  |  |

**Model Summary**

| **S** | **R-sq** | **R-sq(adj)** | **PRESS** | **R-sq(pred)** |
| --- | --- | --- | --- | --- |
| 0.0370810 | 99.31% | 99.06% | 0.02475 | 98.45% |

**Means**

| **Treatments** | **N** | **Mean** | **StDev** | **95% CI** |
| --- | --- | --- | --- | --- |
| c1 | 3 | 1.2100 | 0.0361 | (1.1606, 1.2594) |
| c2 | 3 | 1.0300 | 0.0608 | (0.9806, 1.0794) |
| T1 | 3 | 0.37000 | 0.01000 | (0.32063, 0.41937) |
| T2 | 3 | 0.4400 | 0.0200 | (0.3906, 0.4894) |

*Pooled StDev = 0.0370810*

**Fisher Pairwise Comparisons**

**Grouping Information Using the Fisher LSD Method and 95% Confidence**

| **Treatments** | **N** | **Mean** | **Grouping** | | | |
| --- | --- | --- | --- | --- | --- | --- |
| c1 | 3 | 1.2100 | A |  |  |  |
| c2 | 3 | 1.0300 |  | B |  |  |
| T2 | 3 | 0.4400 |  |  | C |  |
| T1 | 3 | 0.37000 |  |  |  | D |

*Means that do not share a letter are significantly different.*
